# Integrated mRNA and microRNA transcriptome variations in the multi-tepal mutant provide insights into the floral patterning of the orchid *Cymbidium goeringii*

**DOI:** 10.1186/s12864-017-3756-9

**Published:** 2017-05-11

**Authors:** Fengxi Yang, Genfa Zhu, Zhen Wang, Hailin Liu, Qingquan Xu, Dan huang, Chaoyi Zhao

**Affiliations:** 1 Guangdong Key Laboratory of Ornamental Plant Germplasm Innovation and Utilization, Environmental Horticulture Research Institute, Guangdong Academy of Agricultural Sciences, Guangzhou, 510640 People’s Republic of China; 2 Guangdong Key Laboratory of Biotechnology for Plant Development, College of Life Science, South China Normal University, Guangzhou, 510631 People’s Republic of China

**Keywords:** *Cymbidium goeringii*, Multi-tepal mutant, Floral transcriptome, MicroRNA, Floral patterning, MiR396

## Abstract

**Background:**

*Cymbidium goeringii* is a very famous traditional orchid plant in China, which is well known for its spectacular and diverse flower morphology. In particular, the multi-tepal mutants have considerable ecological and cultural value. However, the current understanding of the molecular mechanisms of floral patterning and multi-tepal development is limited. In this study, we performed expression profiling of both microRNA (miRNA) and mRNA from wild-type and typical multi-tepal-mutant flowers of *C. goeringii* for the first time, to identify the genes and pathways regulating floral morphogenesis in *C. goeringii.*

**Results:**

Total clean reads of 98,988,774 and 100,188,534 bp were obtained from the wild-type and mutant library, respectively, and *de novo* assembled into 98,446 unigenes, with an average length of 989 bp. Among them, 18,489 were identified as differentially expressed genes between the two libraries according to comparative transcript profiling. The majority of the gene ontology terms and Kyoto Encyclopedia of Genes and Genomes pathway enrichment responses were for membrane-building and ploidy-related processes, consistent with the excessive floral organs and altered cell size observed in the mutant. There were 29 MADS-box genes, as well as a large number of floral-related regulators and hormone-responsive genes, considered as candidates regulating floral patterning of *C. goeringii*. Small RNA sequencing revealed 132 conserved miRNA families expressed in flowers of *C. goeringii*, and 11 miRNAs corresponding to 455 putative target genes were considered to be responsible for multi-tepal development. Importantly, integrated analysis of mRNA and miRNA sequencing data showed two transcription factor/microRNA-based genetic pathways contributing to the multi-tepal trait: well-known floral-related miR156/SPL and miR167/ARF regulatory modes involved in reproductive organ development; and the miR319/TCP4–miR396/GRF regulatory cascade probably regulating cell proliferation of the multi-tepal development.

**Conclusions:**

Integrated mRNA and miRNA profiling data provided comprehensive gene expression information on the wild-type and multi-tepal mutant at the transcriptional level that could facilitate our understanding of the molecular mechanisms of floral patterning of *C. goeringii*. These data could also be used as an important resource for investigating the genetics of floral morphogenesis and various biological mechanisms of orchid plants.

**Electronic supplementary material:**

The online version of this article (doi:10.1186/s12864-017-3756-9) contains supplementary material, which is available to authorized users.

## Background

*Cymbidium goeringii* Rchb. f., which belongs to the subgenus *Jensoa* of the genus *Cymbidium*, is one of the most horticulturally important and popular ornamental plants in the orchid family (Orchidaceae) [1]. It blooms in the winter from January to March and is fragrant. In China, *C. goeringii* is called the spring orchid and is regarded as a Spring Festival flower, and holds a strong position in the traditional flower market [1, 2]. As a typical ornamental plant, *C. goeringii* is characterized by highly specialized reproductive strategies and extremely diversified flowers [3–5], and commercially plays a very important role in world flower markets, especially in Japan, Korea and Southeast Asia. Various mutations occur frequently in this orchid family, which greatly diversify the floral morphology and provide substantial commercial value. However, functional genomic studies and the gene discovery associated with floral pattern regulation remains greatly limited in *C. goeringii* [6].

Floral patterning, in terms of floral organ number, arrangement and initiation timing, has been well studied, especially in *Arabidopsis thaliana* and *Antirrhinum majus* [7–9]. Genes controlling floral organ identity have been identified through the genetic analysis of homeotic mutants, leading to the ABCDE model, in which five classes of regulatory genes (A, B, C, D and E) work in a combinatorial manner to confer the organ identities of the four whorls [10]. Among them, class A genes, such as *APETALA1* (*AP1*) in *Arabidopsis*, specify the outer-most floral organs, the sepals. Class B genes, such as *APETALA3* (*AP3*) and *PISTILLATA* (*PI*), specify petals and male organs in concert with class A and C genes, respectively. Class C genes, such as *AGAMOUS* (*AG*), specify the inner-most floral organs, the carpels. The action of the class A, B and C genes is necessary for the specification of organ identity, and mutations in these genes produce homeotic transformations of one organ type into another. For example, the flowers of *Arabidopsis ag* mutants have no stamen or carpel, and lose the ability to terminate meristematic activity. As a result, the mutant generates multi-petal flowers, which have a repeated structure of sepal-petal-petal, including tens of petals [11, 12].

Although the ABCDE model is applicable to most flowering plants, in orchids it shows modifications in the expression domains of the class B *AP3* and *PI-like* genes and of the class C and E genes. The ‘orchid code’ model derived by Mondragon-Palomino and Theissen revealed that orchids typically have four *AP3/DEF-like* genes, representing the ancient gene Clades 1, 2, 3 and 4. With high levels of Clades 1 and 2, and low levels of Clades 3 and 4, gene expression specifies inner lateral tepals, whereas lip development requires low levels of Clades 1 and 2 expression and high levels of Clades 3 and 4 expression [13, 14]. Similarly, the orchid P-code model, which was derived primarily from *Oncidium* orchids, indicates that the higher-order heterotetrameric sepal/petal complex (OAP3-1/OAGL6-1/OAGL6-1/OPI) specifies sepal/petal formation, whereas the lip complex (OAP3-2/OAGL6-2/OAGL6-2/OPI) is exclusively required for lip formation [15]. However, beyond the hypothetical model of orchid code, we do not know exactly which genes are responsible for the subsequent floral organ specification or the mechanism underlying morphological diversity during orchid flower development. Compared with other plants, few reports have investigated the multi-tepal development of orchids [16]. Especially for the genus *Cymbidium*, available genomic resources are limited. This genetic data is insufficient for elucidating the molecular mechanism of floral regulation in *C. goeringii* [17–19].

Despite the genetic regulation of floral patterning, microRNAs (miRNAs)—which are small (18–25 nt) non-coding RNAs that control the epigenetic regulation of gene expressions— play crucial roles in different processes, from leaf and root morphogenesis to floral induction, organ formation, reproduction and stress response [20, 21]. They originate from single-stranded precursors (pre-miRNAs) able to self-pair and form hairpin structures. In plants, the Dicer1-like RNAse III enzyme excises the miRNA/miRNA* duplex from the pre-miRNA [22]. Subsequently, the guide strand of the duplex is recruited by an RNA-induced silencing complex, whose core component is the protein ARGONAUTE1. This complex permits the interaction between the miRNA and its target mRNA, thereby regulating its expression through mRNA cleavage, translational repression, or epigenetic modifications [22, 23]. In recent years, advances in next-generation sequencing techniques have prompted a plethora of studies on miRNAs, in both model and non-model species, and have led to the development of specific *in silico* analysis tools. Increasing numbers of studies on non-model plant species have highlighted the evolutionary conservation of a large number of miRNA families and the existence of taxon-specific ones. Recently, a few studies have also examined miRNAs in orchids, which are characterized by highly diversified floral structures and pollination strategies. Examples such as *Phalaenopsis aphrodite*, *Erycina pusilla* and *Dendrobium officinale* belonging to the sub-family *Epidendroideae* have been sequenced [24–27]. Both conserved and novel miRNAs have been identified in these species. By comparison, miRNA studies in the genus *Cymbidium* are very limited [28].

Here, we examined a typical multi-tepal mutant of *C. goeringii*, the cultivar ‘Yuhudie’, which continues to produce sepals and petals in the inner whorl. Compared to the wild-type plant, the gynostemium is replaced by a new emerged flower and both the lips and gynostemium are misshapen in ‘Yuhudie’. From the computational analysis of the floral mRNA and miRNA expression levels based on deep sequencing data, we identified 98,446 unigenes and 226 conserved miRNAs that were expressed in flower. In total, 18,489 differentially expressed genes (DEGs) and 11 miRNA families corresponding to 455 putative target genes were probably related to the multi-tepal mutation. On this basis, we found a great number of floral-related and plant hormone-responsive genes contributing to multi-tepal development. Importantly, integrated analysis of mRNA and miRNA sequencing data showed that the transcription factor/microRNA-based genetic pathways—including floral-related miR156-SPL and miR167-ARF regulatory modes as well as the miR319/TCP4–miR396/GRF regulatory cascade—were involved in multi-tepal development. These results shed light on the regulatory mechanism of multi-tepal development of *C. goeringii*, and provide a base for future genome-wide orchid biology and biotechnology research.

## Results

### Morphology of the *C. goeringii* multi-tepal mutant

**Fig. 1:**
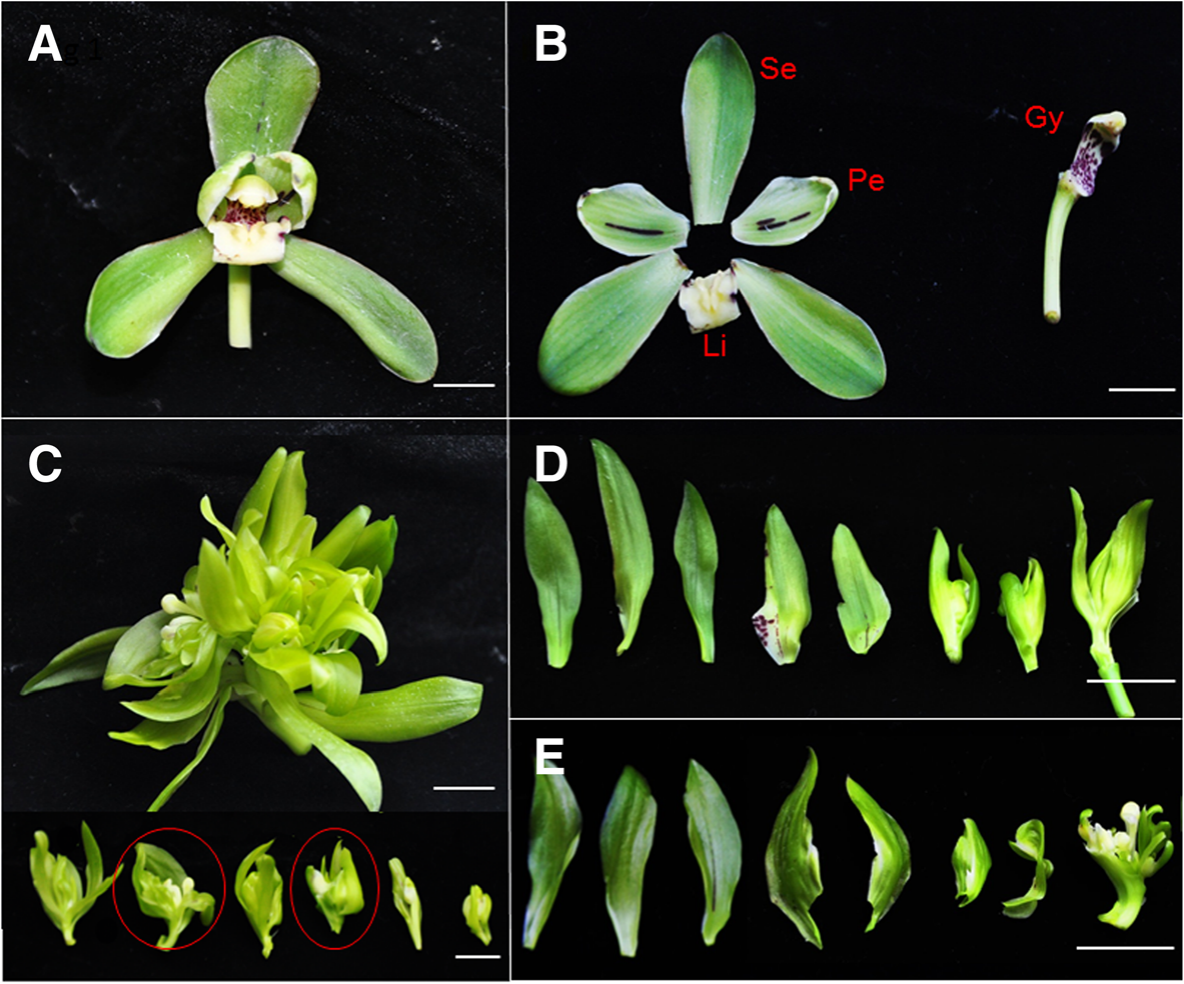
Flower morphology of *C. goeringii* wild-type plant ‘Songmei’ and the multi-tepal mutant ‘Yuhudie’. **a** & **b**: Wild-type flower with three sepals and three petals. Two petals are similar to each other; the third is highly evolved, with an ovate to triangular shape, and is known as the labellum or lip. The male and female reproductive organs are highly fused to form a gynostemium. **c**: The gynostemium is replaced by newly emerged flowers in the multi-tepal mutant, and this ectopic flower continues to produce sepals and petals centripetally. **d** & **e**: Detailed compositions of two of the newly emerged flowers (indicated with *red circle* in **c**). The lips are misshapen; the gynostemia are fused to the margin of the sepals and lack an organized four-pollinia structure. Bar = 1 mm. Se, sepal; Pe, petal; Li, lip; Gy, Gynostemium

The wild-type flowers of *C. goeringii* have three sepals in the first whorl and three petals in the second whorl. Together these are called the tepals. Two of the petals are similar to each other and resemble unmodified sepals, while the third is highly evolved, with an ovate to triangular shape, and it is called the labellum (or lip). The male and female reproductive organs are highly fused to form a gynostemium, which evolved through the complete fusion of the style, stigma and staminal filament, and has four pollinia on a semi-circular viscidium (Fig. 1a & b). In comparison, the mutant has new flowers instead of a single gynostemium in the center, and continues to produce sepals and petals centripetally. Although they are morphologically similar to those of the wild-type, these newly emerged outgrowths are considerably smaller (Fig. 1c–e). The normal sepal and petal have diameters of ~1.23 and ~1.31 cm, respectively, and the total length of them ranges from ~2.78 to ~3.68 cm, whereas the newly emerged sepal and petal in the mutant are only 0.52–0.95 cm in diameter and the outgrowth’s lengths range from ~1.08 to ~3.38 cm (Table 1). On average, mutant petals are 28% shorter and almost 50% narrower than wild-type petals at anthesis. This difference in size is mainly due to a difference in cell size between them. The mutant shows about 30% smaller cell diameter than that of the wild-type (Additional file 1). Moreover, the lips are misshaped in the mutant, and the gynostemia are also mostly fused to the margin of the sepals and lack an organized four-pollinia structure (Fig. 1b & e).

**Table 1:** Morphological phenotype of the multi-tepal flowers. Mature flowers are characterized and the data of each sample was the means ± SD from 20 plants. The experiments were repeated three times with similar results

Genotype	Sepal width (mm)	Sepal length (mm)	Petal width (mm)	Petal length (mm)
Wild type	1.23 ± 0.12	2.78 ± 0.18	1.31 ± 0.10	3.68 ± 0.22
Mutant	0.52 ± 0.08	1.08 ± 0.08	0.95 ± 0.12	3.38 ± 0.16

### Transcriptome sequencing, *de novo* assembly and functional annotation

To determine the probable cause of the multi-tepal mutations and identify the genes associated with floral patterning, two cDNA libraries were constructed for the wild-type and multi-tepal flowers of *C. goeringii*. By sequencing on the Illumina HiSeq 2500 platform, 98,988,774 and 100,188,534 paired-end reads were obtained for wild-type and multi-tepal libraries, respectively. After filtering, and removing the low quality reads, reads containing adapters and reads containing unknown nucleotides (greater than 5%), the clean reads were pooled together and *de novo* assembled into 98,446 unigenes with a mean length of 989 bp, a N50 length of 1519 bp and a total length of 97,314,639 bp. Of the putative unigenes, 23,137 were longer than 1000 bp, which represented 19.1% of the total (Table 2). The size distribution of the assembled transcripts and unigenes is shown in Additional file 2.

**Table 2:** Summary of *C. goeringii* floral transcriptome

Type	Wild type	Mutant	Total
Raw reads	98,988,774	100,188,534	
Clean reads	90,503,180	89,623,606	
Unigenes	79,888	94,004	98,446
Residues	90,372,555 bp	103,636,656 bp	97,314,639 bp
Smallest unigene	301 bp	301 bp	
Largest unigene	24,154 bp	37,800 bp	
Average length	1131 bp	1102 bp	989 bp

**Fig. 2:**
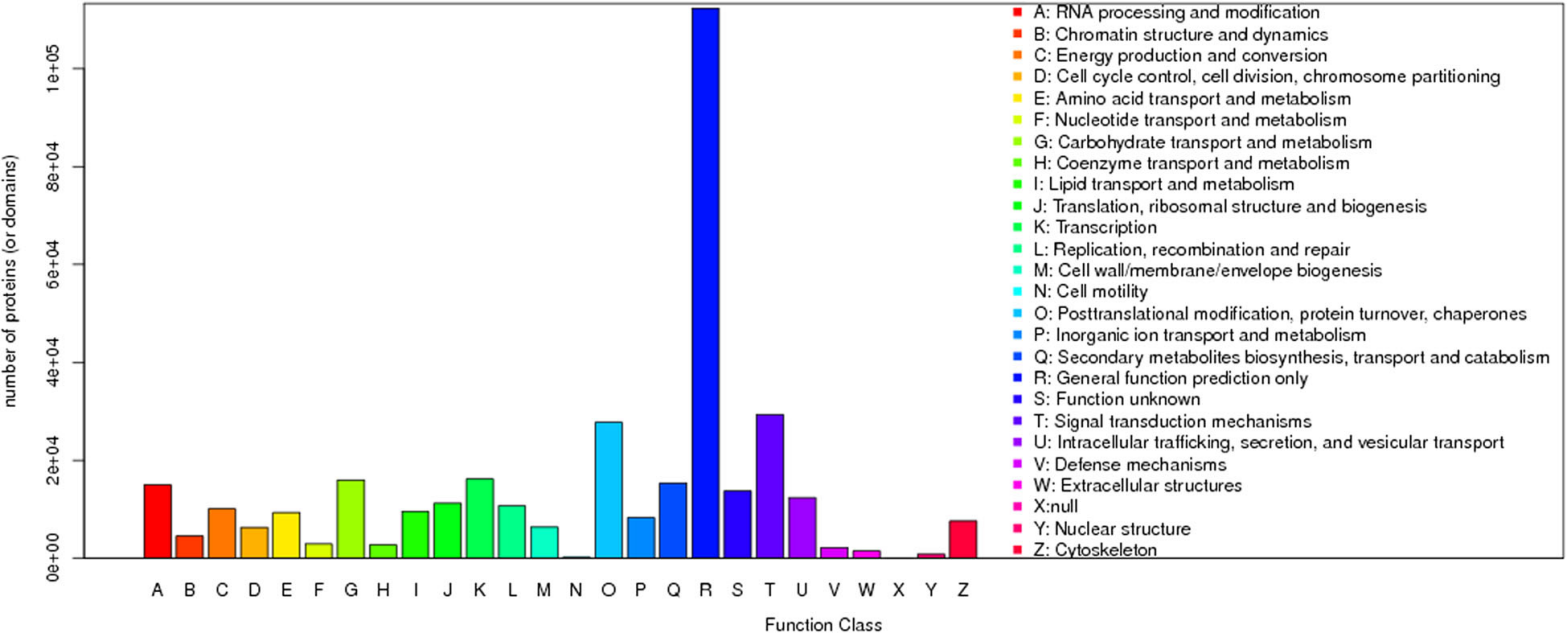
COG function classification of assembled unigenes

The entire set of unigenes was annotated on the basis of their similarities with known or putative annotations in public databases. A total of 78,175 unigenes had at least one significant match with an existing gene model in searches using the BLAST algorithm (Table 3). Thus, more than half of the assembled sequences had putative functional identifications. Among them, 46,342 (47.07%) and 33,966 (34.50%) unigenes were aligned to a protein present in the NCBI non-redundant protein (Nr) and SwissProt databases, respectively. There were 42,200 unigenes (42.87%) classified into 25 functional classifications in Clusters of Orthologous Groups (COG) classification. ‘General function prediction’ was dominant, followed by ‘Post-translational modification’ and ‘Signal transduction’. ‘RNA processing and modification’, ‘Carbohydrate transport and metabolism’, ‘Secondary metabolites biosynthesis’ categories and ‘Transcription’ also shared a relatively high percentage (Fig. 2), which is similar to results for the closely related *C. ensifolium* in our previous study [18].

**Table 3:** Summary of the blast hits against the known protein database

	Number	Percent
Total	78,175	79.40%
Nr	46,342	47.07%
Swiss prot	33,966	34.50%
COG	42,200	42.87%
GO	24,563	24.95%
KEGG	11,875	12.06%

**Fig. 3:**
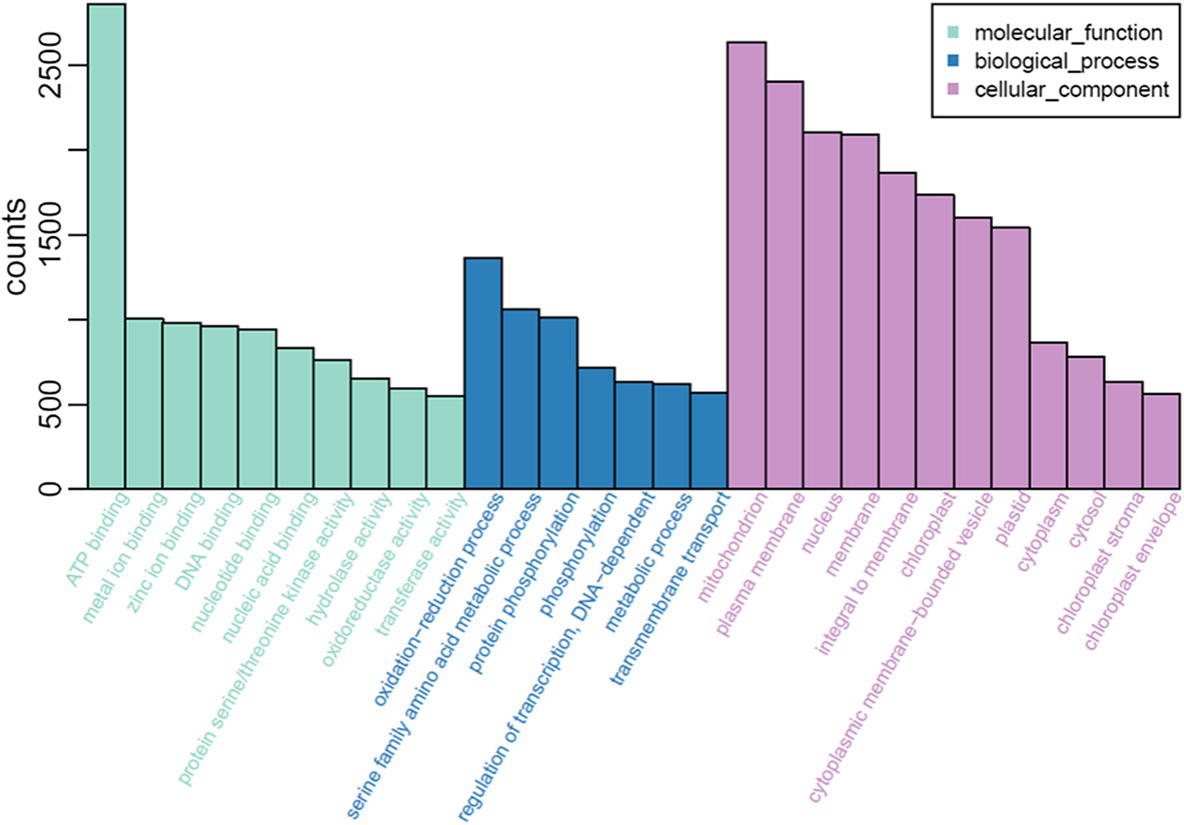
GO classification of assembled unigenes

**Fig. 4:**
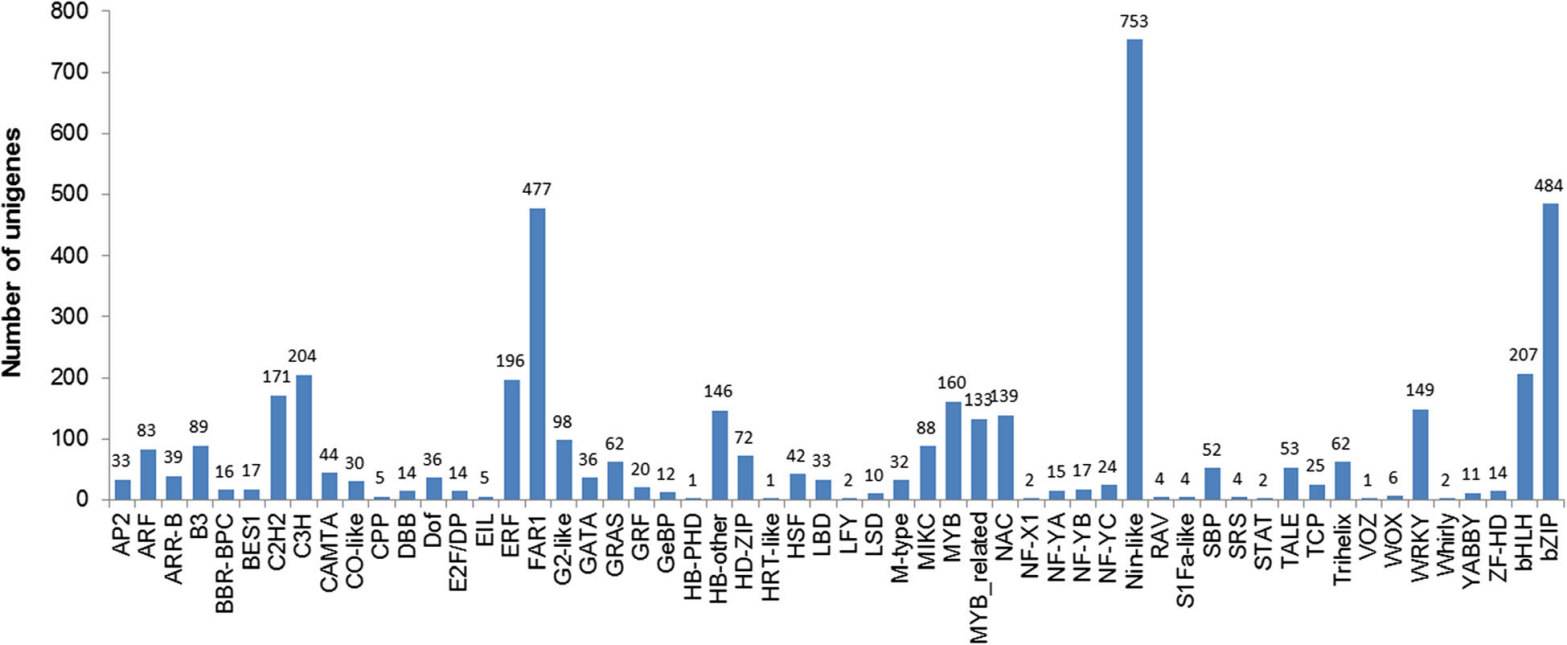
Predicted transcription factors of *C. goeringii*

A total of 24,563 unigenes (24.95%) were assigned to 29 functional groups using Gene Ontology (GO) assignments. The three major categories (biological process, cellular component and molecular function) were assigned 16,859, 16,255 and 18,122 GO terms, respectively. Within each of the three main categories of the GO classification scheme, the dominant subcategories were ‘ATP binding’, ‘oxidation − reduction process’ and ‘mitochondrion’, respectively. ‘Amino acid metabolic process’, ‘protein phosphorylation’, ‘plasma membrane, chloroplast cytoplasmic membrane-bounded vesicle’ were also well represented (Fig. 3). These genes were mainly correlated with ‘binding’, ‘membrane-bounded organelles’ and ‘organelle parts’, which correlated well with the genes identified in the stamen or pollen transcriptomes of other plants. Additionally, 11,875 unigenes were assigned to 331 Kyoto Encyclopedia of Genes and Genome (KEGG) pathways. Of these, the ‘metabolic pathways’ made up the majority, followed by ‘biosynthesis of secondary metabolites’, ‘ribosome’ and ‘cell cycle’. Details of the pathway annotations for significant hits in unigene sets are provided in Additional file 3. We also aligned these unigenes to the Plant Transcription Factor Database (PTFDB) and identified 4451 unigenes as transcription factor sequences belonging to 56 putative transcription factor families (Fig. 4) using the BLASTX algorithm with a cut-off E-value below 10^−5^ (http://planttfdb.cbi.pku.edu.cn/). Thus, the ESTs generated during this study provide a valuable resource for gene discovery and future functional analyses of *C. goeringii*.

### Comparison of transcriptomes between the wild-type and the multi-tepal mutant

We investigated the expression levels of unigenes in the wild-type and the mutant plant by comparing these two libraries using a Fragments Per Kilobase of transcript per Million mapped reads (FPKM) analysis, with a false discovery rate ≤ 0.05 and |log_2_ratio| ≥ 1. Subsequently, 18,489 unigenes were found to be differentially expressed, of which 9619 unigenes were up-regulated and 8871 unigenes were down-regulated in the mutant flower as compared with the wild-type. Among them, 412 unigenes (12.8%) showed over a 10-fold change in expression level (Additional file 4).

**Fig. 5:**
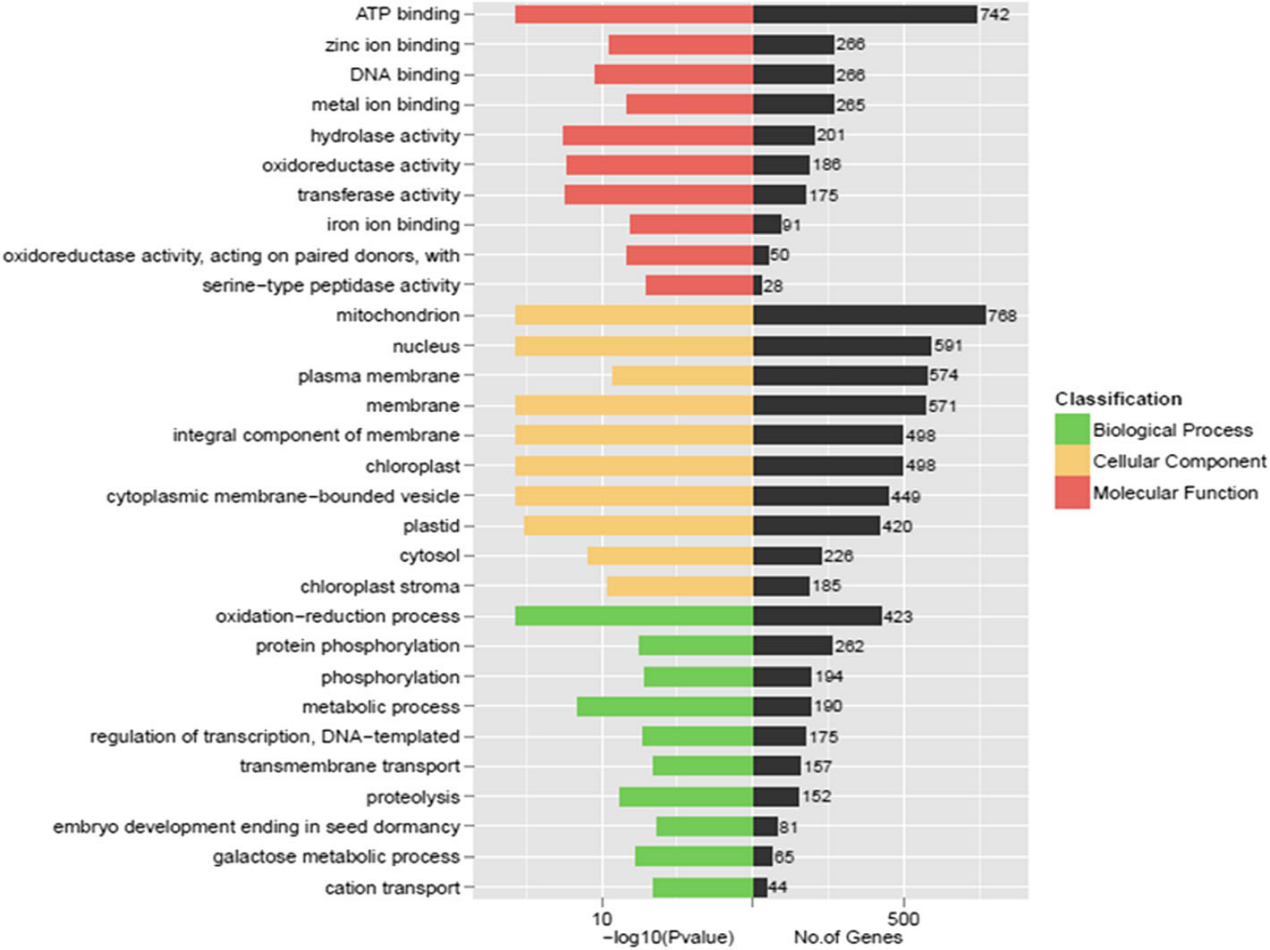
GO classification of unigenes differentially expressed between the wild-type and the mutant

The set of 18,489 common DEGs between the wild-type and mutant was mapped in accordance with the GO biological process database and the KEGG pathway to identify the genes involved in important biological processes. After the GO term enrichment analysis, 8705 genes from the 18,489 DEGs were assigned to the three main categories: ‘biological process’ (1743), ‘cellular component’ (4682) and ‘molecular function’ (2280). The top five DEG-enriched GO terms were ‘mitochondrion’, ‘ATP binding’, ‘nucleus’, ‘plasma membrane’ and ‘membrane’ (Fig. 5). For the pathway enrichment analysis, we mapped those differentially expressed unigenes to terms in the KEGG database and searched for KEGG terms that were significantly enriched compared with the transcriptome background. In total, 4222 DEGs were assigned to 288 KEGG pathways. The pathways that were most represented by the unigenes were ‘RNA transport’ (122), followed by ‘spliceosome’ (118), ‘biosynthesis of amino acids’ (102) ‘starch and sucrose metabolism’ (98), ‘protein processing in endoplasmic reticulum’ (95), ‘mRNA surveillance pathway’ (92) and ‘RNA degradation’ (89) (Additional file 5).

The morphological analysis indicated a significantly decreased cell size of the mutant flower, which was probably the primary cause of the small and narrower petals of the multi-tepal mutant. Considering that the expansion of plant cells necessarily requires a loosening of the cell wall, accompanied by ploidy increase through endoreduplication, with further remodeling of cell walls—including the production and trafficking of polysaccharides and membrane-associated proteins—it is reasonable that the unigenes responding to membrane-building and ploidy increase showed differential expression in the mutant flowers. This included ‘mitochondrion’, ‘plasma membrane’, ‘nucleus’ and ‘RNA transport’ recombination’ (Table S3). These GO terms and KEGG classifications serve as indications of significantly different biological processes occurring in the two different phenotypical flowers, which could offer clues for further studies to determine their functions in multi-tepal development.

### DEGs related to floral development

**Fig. 6:**
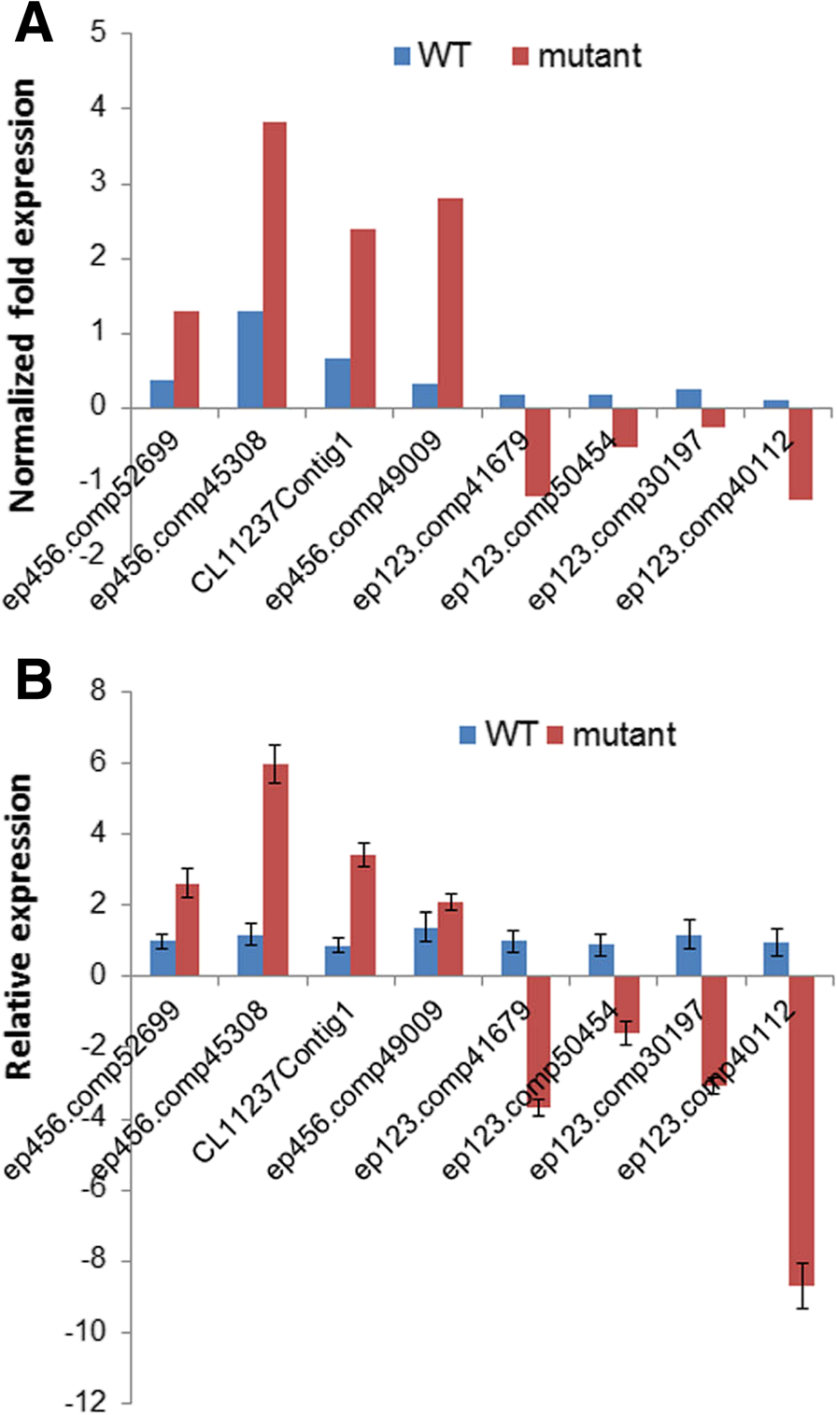
Comparative analysis of transcriptome sequencing based expression data with the real-time RT-PCR expression data. **a**: Normalized fold expression of the MADS-box genes: FPKM value of each individual gene normalized with the putative ubiquitin gene. **b**: real-time RT-PCR analysis of MADS-box gene expression in the wild-type and the mutant (normalized to the expression of *Ubiquitin*). The y-axis indicates fold change in expression among the samples. Error bars indicate the standard deviation of the mean (SD) (*n* = 3). Three replicates were analyzed, with similar results

The identities of flower organs are specified by the interactions of A, B, C, D and E class MADS-box genes. Additionally, the orchid plant has a specific P-code model that modulates floral development, in which the class B gene *AP3*/*DEF-like* determines the identity of the lateral petals and lip, while the class B gene *PI*/*GLOBOSA-like*, along with class A, C, D and E genes, retain their functions. Therefore, we focused on the changes in the floral-related MADS-box genes between the wild-type and mutant, aiming to reveal the candidates associated with multi-tepal development. Based on the reference annotation, we identified 110 putative MADS-box genes (Additional file 6). Among them, 29 unigenes, including *AP1-*related genes (2), *AP3/DEF*-*like* genes (16) and *AGAMOUS*-*like* classes (11), were differentially expressed, with 16 up- and 13 down-regulated in the mutant (Table 4). Eight of them were randomly chosen for further confirmation. Normalized expression values of these genes were created by calculating the value of Fragments Per Kilobase of transcript per Million mapped reads (FPKM) of each individual gene against the FPKM value of *Ubiquitin*, and the real-time RT-PCR expression data were also normalized to the expression of *Ubiquitin*. Results from the real-time RT-PCR assay followed similar trends to those of the read numbers (Fig. 6).

**Table 4:** Differentially expressed MADS-box genes related to floral development. Fold change = log2(expression value of each gene of the mutant/wild-type), expression value = FPKM [total transcript fragments/mapped fragments (millions)] × transcript length (kb)

Sequence ID	Fold change	Putative function
Up-regulated		
ep456.comp45403_c0_seq2	7.24	MADS-box protein 3 [*Cymbidium ensifolium*]
ep456.comp45308_c0_seq2	6.83	AP1-related protein [*Phalaenopsis amabilis*]
ep456.comp59563_c0_seq4	6.51	APETALA3-like protein [*Papaver somniferum*]
ep456.comp58153_c0_seq7	5.79	SOC1 [*Dendrobium hybrid cultivar*]
ep123.comp42801_c0_seq3	5.55	SOC1 [*Dendrobium hybrid cultivar*]
CL11237Contig1	4.58	MADS box transcription factor 1 [*Oncidium hybrid cultivar*]
ep456.comp52699_c0_seq6	3.89	DEFICIENS-like MADS-box transcription factor [*Gongora galeata*]
ep456.comp50057_c0_seq1	2.90	mads box protein, putative [*Ricinus communis*]
ep456.comp50389_c0_seq1	2.67	SOC1 [*Dendrobium hybrid cultivar*]
ep456.comp49009_c0_seq1	2.62	AP3 [*Cymbidium goeringii*]
ep456.comp58153_c0_seq3	2.45	MADS box protein [*Phalaenopsis equestris*]
ep456.comp52699_c0_seq10	2.35	DEFICIENS-like MADS-box transcription factor [*Gongora galeata*]
ep456.comp52699_c0_seq9	2.04	DEFICIENS-like MADS-box transcription factor [*Gongora galeata*]
ep456.comp36865_c0_seq1	3.81	putative MADS-box transcription factor family protein [*Zea mays*]
ep456.comp59687_c2_seq7	2.27	MADS1 protein [*Eschscholzia californica*]
ep456.comp59687_c2_seq1	2.26	MADS1 protein [*Eschscholzia californica*]
Down-regulated		
ep123.comp50454_c0_seq6	−2.12	AGL66 protein [*Eschscholzia californica*]
ep123.comp36586_c0_seq2	−2.72	AP3 [*Cymbidium goeringii*]
ep123.comp44547_c1_seq1	−2.98	MADS box transcription factor [*Elaeis guineensis*]
ep123.comp41679_c0_seq1	−3.45	DEFICIENS-like MADS-box transcription factor [*Gongora galeata*]
ep123.comp30197_c0_seq1	−3.89	C-class MADS-box-like protein [*Orchis italica*]
ep123.comp40112_c1_seq1	−6.16	AGAMOUS-like transcription factor [*Dendrobium crumenatum*]
ep123.comp42801_c0_seq2	−6.60	SOC1 [*Dendrobium hybrid cultivar*]
ep123.comp35467_c0_seq1	−6.63	AP1-like protein [*Cymbidium faberi*]
CL8650Contig1	−6.92	SOC1 [*Dendrobium hybrid cultivar*]
ep123.comp20001_c0_seq1	−7.03	Agamous-like MADS-box protein AGL62 [*Aegilops tauschii*]
ep123.comp42801_c0_seq1	−7.50	SOC1 [*Dendrobium hybrid cultivar*]
ep123.comp41679_c0_seq4	−7.91	DEFICIENS-like MADS-box transcription factor [*Gongora galeata*]
ep123.comp42801_c0_seq4	−8.59	SOC1 [*Dendrobium hybrid cultivar*]

**Fig. 7:**
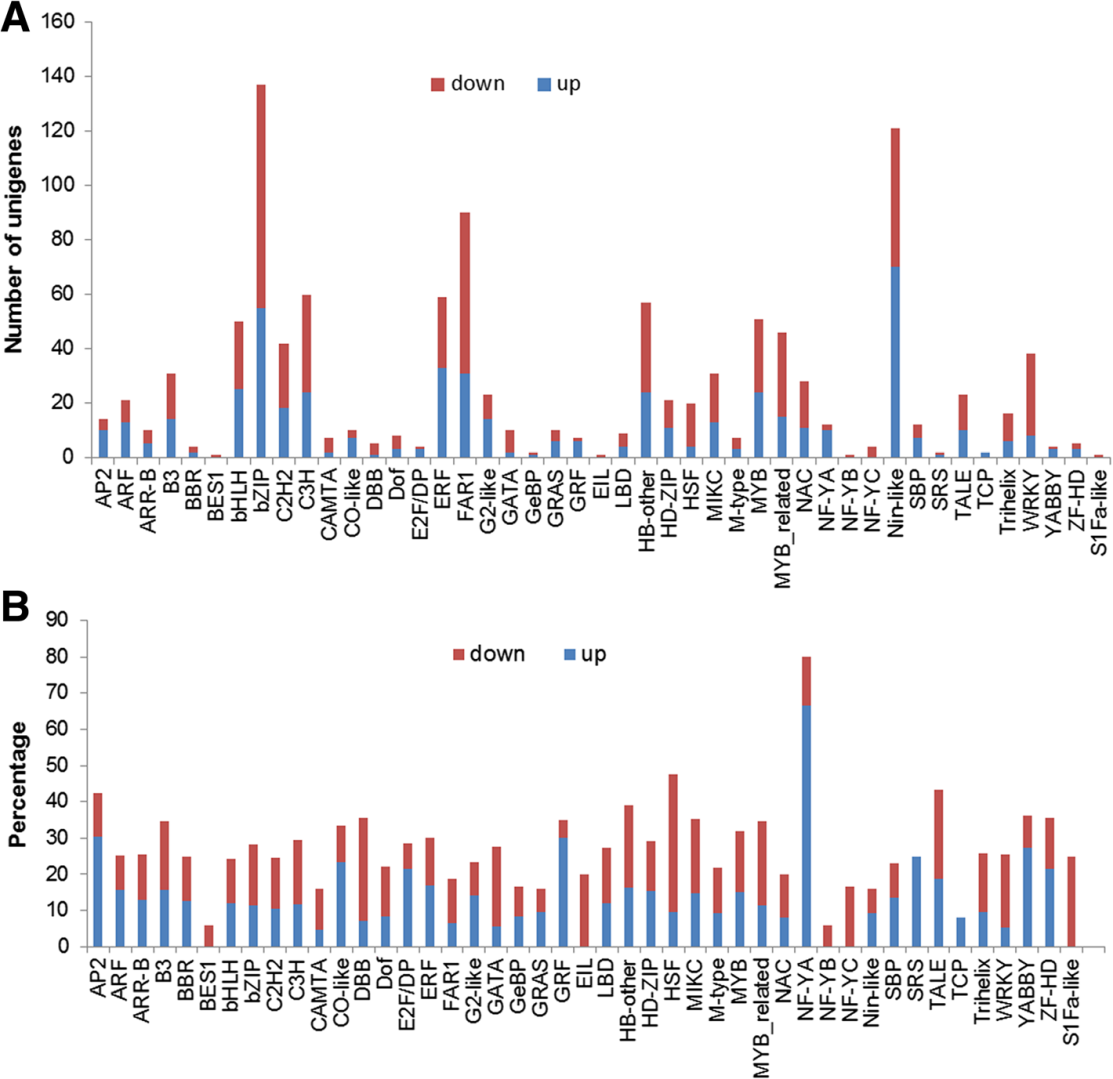
Transcription factors differentially expressed between the wild-type and the mutant. **a**: Number of up-regulated (*blue*) and down-regulated (*red*) transcripts were quantified in the mutant compared to the wild-type (based on the FPMK value with |log_2_(fold change)| > 1 and Q-value < 0.05). **b**: percentage of up- and down-regulated genes after normalization to the total number of family members

In addition to the typical ABCDE genes that related to floral development, a great number of other transcription factors were also differentially expressed, accounting for more than 25% of *C. goeringii* transcription factors. In total, 1118 of 4451 transcription factors showed significantly different expression levels in the mutant, indicating functions associated with the plant floral patterning. There were 504 and 614 transcription factors—including bZIP, FAR1 and Nin-like—specifically up- and down-regulated in the mutant compared to the wild-type, respectively (Fig. 7a). After normalization to the total number of family members, we found that most differences occurred in the transcription factor family of AP2, NF-YC, TALE and HSF (more than 40% of the members were differentially expressed) (Fig. 7b), suggesting a crucial role for them in *C. goeringii* floral development. For example, percent AP2 transcription factors act primarily in the regulation of developmental programs in *Arabidopsis—AP2-13* (*AP2*), *AP2-05* (*AINTEGUMENT*, *ANT*) and *AP2-09* (*ANT-LIKE1*) genes regulate floral growth and ovule development [29, 30]. NF-Y interacts with CONSTANS in the photoperiod pathway and DELLAs in the gibberellin pathway. This CO/NF-Ys complex further regulates the transcription of *SOC1*, a major floral pathway integrator [31]. TALE genes are also required for meristem maintenance and proper patterning of organ initiation, and play a critical role in the diversity of flower and leaf form [32]. All of these new *de novo* transcriptome data sets provide a significant resource for the discovery of genes related to floral patterning and improve our understanding of the regulation of multi-petal mutants. However, the roles of these transcription factors in multi-tepal development and floral patterning require further investigation.

### DEGs related to hormone response involved in floral development

**Fig. 8:**
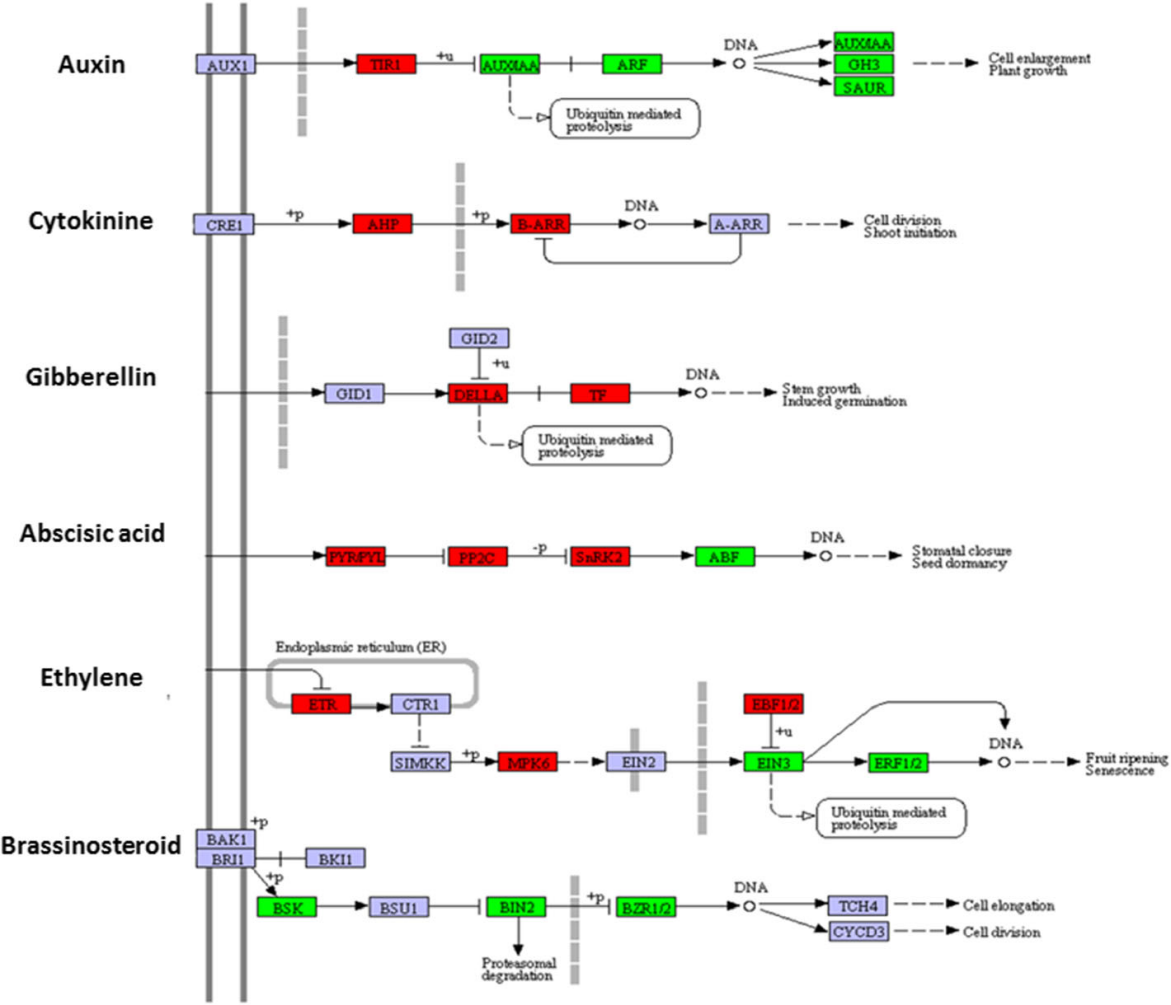
DEG-enrichment of the plant hormone signal transduction pathway by KEGG annotation. The key regulatory components in multiple hormone response pathways are presented as their names (*red*, up-regulated; *green*, down-regulated). Gene IDs and fold changes in transcript abundance are indicated in Additional file 4

Plant hormones play important roles in floral development, and their signaling pathways in plants are interconnected in a complex network to regulate flower organ primordia formation, organ specification and final organ size. Comparative analysis of transcriptomes between wild-type and mutant showed a significant enrichment of the plant hormone signal transduction pathway with a P-value of 8.6E-21, and with 70 unigenes involved (Fig. 8 and Additional file 7). Among them, auxin exerts its regulatory role, at least to an extent, by controlling the fundamental processes of cell division, expansion and differentiation. The ortholog of the gene encoding the F-box protein TRANSPORT INHIBITOR RESPONSE1 (TIR1), an auxin receptor, showed altered expression levels with increases in transcript abundance. Another three well-known groups of early auxin-responsive genes [33] including the auxin/indole-3-acetic acid (Aux/IAA), the small auxin up RNA (SAUR) and the auxin-responsive Gretchen Hagen3 (GH3) gene families were generally repressed in the mutant. For example, the decrease of auxin-responsive protein IAA-like genes, ep123.comp37580/comp42487/comp50482/comp47687/comp42487, ranged from 6.8- to 2.2-fold, and the expressions of SAUR and GH3 family genes also decreased by 5.0- to 2.3-fold in the mutant (Additional file 7).

In addition to the auxin-associated genes, notable differential expressions were also observed among the cytokinin-responsive genes. For example, expressions of the hybrid histidine protein kinases known as cytokinin receptors [34], histidine phosphotransfer proteins (AHPs) [35] and nuclear response regulators (ARRs) that serve as transcriptional regulators in the cytokinin two-component signaling pathway [36] were also largely up-regulated, ranging from 3.8- to 5.4-fold, suggesting a positive regulation of cytokinin in multi-tepal development.

Genes involved in responses to other hormones, such as gibberellin, abscisic acid and ethylene, were also highly enriched. The homology levels of the DELLA family genes *GA INSENSITIVE* (*GAI*) and *RGA-LIKE1* (*RGL1*) [37] indicated 2.1- to 4.8-fold change in the mutant (Additional file 7). DELLA proteins inhibit growth first by altering the cell division rate during the proliferation phase, followed by altering the cell expansion rate during the expansion phase. A genetic analysis in *Arabidopsis* showed that the DELLA proteins RGA and RGL2 jointly repress petal, stamen and anther development in gibberellin-deficient plants, and that this function is enhanced by RGL1 activity [38, 39]. In our study, the expression of DELLA family genes and downstream responsive transcription factors significantly altered, which revealed a critical role of gibberellin in floral patterning regulation. Abscisic acid-related genes such as the *PYR/PYL/RCAR* family of receptors, 2C-type protein phosphatases, SnRK2-type kinases and downstream abscisic acid responsive element-binding factors [40, 41] were also greatly altered in the mutant. These results suggest important roles for the hormone pathway in floral patterning regulation, and all candidate genes provided important clues concerning multi-tepal development.

### Small RNA profiling in wild-type and mutant *C. goeringii* flowers

Despite the numerous mRNAs involved in floral development, miRNAs, which regulate mRNA expressions at the post-transcriptional level, also play essential roles in floral organ identity. To investigate the effects of miRNAs during multi-tepal development in *C. goeringii*, we constructed two small RNA libraries from the wild-type and the mutant flowers, independently. Deep sequencing of the small RNA libraries generated 14,263,624 and 15,042,951 raw reads, respectively, ranging from 17 to over 30 nucleotides in length, and the most represented lengths were 20–23 nt (Additional file 8). After removing adaptor, insert and poly(A) contaminations and low quality reads (smaller than 17 nt), 10,142,278 clean reads from wild-type and 10,400,447 from the mutant were obtained, among which 9,292,059 (91.6%) and 9,611,868 (92.4%), respectively, unique reads mapped to our transcriptome dataset. The mapped small RNA sequences were clustered into several RNA classes, including conserved miRNAs, transfer RNA (tRNA), ribosomal RNA (rRNA), small nuclear RNA (snRNA), small nucleolar RNA (snoRNA), Piwi-interacting RNA (piRNA) and an uncharacterized group (Table 5). Approximately 6.29% and 6.51% of the unique reads from wild-type and mutant, respectively, were annotated as miRNA.

**Table 5:** Summary of small RNA sequencing data

Type	WT	Count %	MU	Count %
Clean reads	10,142,278	100%	10,400,447	100%
Mapped reads	9,292,059	91.6%	9,611,868	92.4%
miRNA	637,796	6.29%	677,295	6.51%
tRNA	232,530	2.29%	257,376	2.47%
rRNA	3,255,427	32.10%	3,542,808	34.06%
snRNA	79,518	0.78%	88,029	0.85%
snoRNA	209,573	2.07%	280,213	2.69%
piRNA	32,936	0.32%	35,682	0.34%
Y_RNA_etc	5,694,498	56.15%	5,519,044	53.08%

To identify conserved miRNAs in *C. goeringii*, all of the mapped small RNA reads in the transcriptome were used as query against known miRNAs in the database. In total, 226 conserved miRNAs, representing 132 known miRNA families were obtained. Of these, 92 from wild-type and 94 from mutant were perfectly matched to known miRNA families of other plant species, respectively. The distribution of miRNAs in different miRNA families is shown in Table 6 and Additional file 9. The most abundant miRNA was the miR159 family, accounting for 38.6 and 21.6% of the total miRNA in the wild-type and mutant, respectively. Several other miRNA families, such as miR319, miR162 and miR166 families, also had high abundance of expression, consistent with the previous report of the floral miRNA expression profiling in *P. aphrodite* and *C. ensifolium*. Interestingly, we also found relatively high expressions of the miR396, miR6478 and miR2916 families both in the wild-type and the mutant flower, indicating their putative involvement in floral pattering of *C. goeringii.*

**Table 6:** The top 25 miRNA families expressed at highest levels in *C.goeringii*. miRNA expression levels were calculated by RPM value. RPM (reads per million) = number of reads mapping to miRNA number of reads in clean data × 10^6^

	Wild-type	Mutant
miR159	6302.99	6165.40
miR319	1307.90	5413.10
miR396	559.20	2902.70
miR162	716.16	741.55
miR6478	703.50	690.90
miR166	347.07	1274.07
miR2916	465.28	719.95
miR172	249.73	326.75
miR171	204.25	140.29
miR902l	128.64	121.44
miR167	87.83	415.08
miR390	103.38	72.31
miR535	88.06	241.98
miR398	284.38	44.62
miR6300	33.59	38.61
miR528	3.84	17.58
miR164	29.08	24.02
miR168	28.56	32.16
miR156	27.50	125.41
miR5077	25.30	45.50
miR100	22.95	13.56
miR6173	17.42	8.02
miR9760	10.49	11.25
miR5368	10.38	7.89
miR5083	9.54	36.30

In addition to the conserved miRNAs, we identified novel miRNAs in *C. goeringii* flowers. In total, 28 new miRNA candidates from 25 predicted miRNA precursors were obtained, corresponding to 685 unique RNA sequences (Table 7). The miRNA* of these novel miRNAs were also predicted, providing more evidence that they were indeed novel miRNAs of *C. goeringii*. The precursor lengths of the new miRNAs ranged from 74 to 228 nt, with an average length of 128 nt. The average minimum free energy was −38.3 kcal mol^−1^, with a range from −81.9 kcal mol^−1^ to −20.6 kcal mol^−1^. All of the predicted precursors could fold into a characteristic stem-loop structure with the mature miRNA on either the 5′ arm or the 3′ arm of the precursor.

**Table 7:** *C. goeringii* novel miRNAs and their precursors identified in this study

miRNA	Sequence	Read number	Chr-location	Length (nt)	Potential_energy (kcal/mol)
Cgo-m0001-3p	AGGCGGCGCGUGCGGGUUGG	11	CL4439Contig1:400:473:+	74	−30.60
Cgo-m0001-5p	GAGCUUGUGUGGCACAAAAG	32	CL4073Contig1:867:932:+	66	−21.40
Cgo-m0002-5p	CGUGAGCCAGGGCAUCAAGCA	10	ep123.comp16229_c0_seq1:56:148:-	93	−24.70
Cgo-m0003-5p	CAUGCUGGUUCAGCGGCGUCG	76	ep456.comp50928_c0_seq1:277:367:+	91	−30.00
Cgo-m0004-3p	AUCGAAUGUGUAGGAUAGGUGGG	10	ep123.comp41527_c0_seq11:1016:1092:-	77	−24.20
Cgo-m0004-5p	CUUUGGGCCUUUCCUGCGCAGC	50	ep123.comp41527_c0_seq13:1138:1220:-	83	−42.00
Cgo-m0005-5p	CCGGAAGGUCAAGGAAGUUGG	57	ep123.comp41527_c0_seq13:1397:1476:-	80	−37.30
Cgo-m0006-3p	CACCUUGACUAUAGCUUCGC	13	ep123.comp41527_c0_seq8:1017:1091:-	75	−24.20
Cgo-m0006-5p	UUCUGGCGAAGGCUCUUCAGU	18	ep123.comp41527_c0_seq1:1797:1877:-	81	−26.70
Cgo-m0007-3p	AUCGAAUGUGUAGGAUAGGUGG	53	ep123.comp41527_c0_seq4:1017:1091:-	75	−24.20
Cgo-m0008-3p	CUUCUUUUAACUCUACUGAUG	12	ep123.comp45914_c0_seq1:1353:1426:+	74	−21.40
Cgo-m0010-3p	CAGUUGGAGAGUUUGGCUGU	12	ep456.comp295757_c0_seq1:267:343:-	77	−23.60
Cgo-m0010-5p	UAUCGCGUAUUUCAGACUGUG	20	ep123.comp48991_c0_seq1:1338:1424:+	87	−21.80
Cgo-m0011-3p	GGAGAAGGAAGGUGGUCAUGGU	15	ep456.comp48451_c0_seq2:257:343:-	87	−30.80
Cgo-m0012-5p	AUUUUUAGCGCGGAUUCUGACU	10	ep456.comp48041_c0_seq1:1552:1628:+	77	−20.60
Cgo-m0013-3p	GGAGAAGGAAGGUGGUCAUGG	30	ep456.comp48451_c0_seq2:258:342:-	85	−30.80
Cgo-m0013-5p	GAAUUGGUCGACUCAUCAGG	10	ep456.comp53024_c0_seq1:6547:6616:+	70	−24.60
Cgo-m0014-5p	GCGCGCAGUGAACUGGUUUUCUG	26	ep456.comp53024_c0_seq5:3619:3713:+	95	−29.80
Cgo-m0015-5p	GCCGGAAGGUCAAGGAAGUUGGUG	80	ep456.comp53024_c0_seq5:5411:5491:+	81	−37.60
Cgo-m0016-5p	UUUGCAGGAGUUAUGUAUCGG	21	ep456.comp52937_c0_seq1:286:375:+	90	−25.30
Cgo-m0017-3p	CGGAUUUAUGCCGGACGCAGCU	27	ep456.comp53646_c0_seq2:117:189:-	73	−27.10
Cgo-m0017-5p	AUGCUUCAUCAUCUGGUCGUUGA	12	ep456.comp55956_c0_seq1:1633:1732:-	100	−20.80
Cgo-m0018-5p	AUGGCCUGAUAUUGCAAUUG	30	ep456.comp57349_c0_seq1:186:273:+	88	−29.30
Cgo-m0019-5p	AUGGCCUGAUAUUGCAAUUGCGUG	40	ep456.comp57349_c0_seq3:186:273:+	88	−29.30
Cgo-m0020-3p	UGGGAACCUGAAAGGUGGUGG	10	ep456.comp58210_c0_seq1:4180:4271:-	92	−26.40

### Target prediction and functional analysis of miRNA in *C. goeringii* flowers

We used two approaches to identify miRNA targets in *C. goeringii* as described by Chao et al. (2013)[26] (see target prediction procedure section). The first approach, based on extensive complementarity between plant miRNAs and their targets, predicted 5004 miRNA target transcripts for 132 known miRNA families in *C. goeringii* without relying on known targets of other organisms, which resulted in 3085 targets from wild-type and 4254 from the mutant. The second approach relies on the target homology of the previously reported targets of known miRNAs in *Arabidopsis*, and resulted in 3099 predicted target genes in 46 miRNA families (Additional file 10). Of the predicted miRNA targets, 1644 target genes from 12 conserved miRNA families were identified by both methods.

The truncated ESTs in our transcriptome limited the target prediction of the first approach because it relies on the direct alignment of miRNAs to EST sequences, while the second approach searched only for homologs, not for orchid-specific targets. Given that each of the approaches has its own strengths and limitations, using both homology-dependent and homology-independent approaches resulted in a more comprehensive target identification than using either one alone, especially for non-model organism lacking complete transcriptome sequence data.

The total of 6459 miRNA target unigenes were used as query against the Nr database, with 5089 hits that exceeded the E-value threshold. Among them, 64 were involved in floral development/flowering time (indicated by red in Additional file 11). Examples include the floral homeotic protein APETALA 2 (miR172), AP3/MADS5/DEFICIENS-like MADS-box transcription factor (miR156 and miR5179) and many other floral-related transcription factors, including TCP, MYB and SPL. Some are overlapping targets that resulted from miRNA sequence similarities. For example, miR156 and miR535 have overlapping predicted target sites in the SPL family of transcription factors. MiR159 and miR319 have five overlapping predicted targets, including two MYB genes and three kinase genes involved in post-translational modification. These results are consistent with observations in *Arabidopsis* and suggest that the expression of some mRNAs may be regulated by coordinated actions of multiple miRNAs in *C. goeringii*. Additionally, 29 targets involved in various signaling pathways, such as ARF (targeted by miR160 and miR167), NAC (targeted by 164), NF-YA (targeted by miR169), GRF (targeted by miR396) and HD-ZIP III transcription factors (targeted by miR165/166) were also considered to be associated with floral development (indicated by red in Additional file 11). However, a large number of targets were only annotated with vague information, such as 95 targets that were ‘unnamed protein’ and 93 that were not predicted.

### Differentially expressed miRNAs in the multi-tepal mutant and their target genes

Normalized miRNA levels were compared between the wild-type and the mutant to identify the differential expression of miRNAs, and miRNAs that had a fold change log2 > 1 or < −1, and *P* < 0.05, were considered to be differentially expressed. Subsequently, 11 differentially expressed miRNAs were obtained in the mutant, with only two miRNAs being down–regulated, while nine miRNAs were up-regulated. 455 of their corresponding annotated targets were also obtained—some of them are listed in Table 8. Among them, the conserved miRNA families, miR319 and miR396, were more than two-fold greater in the mutant. Ten of their putative target mRNAs presented correlated expression changes, with seven showing a positive correlation and three showing a negative correlation. Examples included the target genes of miR396 and miR319, ep456.comp59964 (GRF) and ep123.comp31487 (TCP), respectively, which increased by more than eight- and four-fold, whereas their targeted mRNAs, ep456.comp55669 and CL10849Contig1, respectively, were down-regulated. Other floral-related miRNA families (miR156, miR166 and miR167) were approximately two-fold higher in the mutant. Of the targeted mRNAs, 13 were found to be correspondingly differentially expressed between the wild-type and the mutant. Among them, the putative target genes of miR156, SPL-like transcription factors ep456.comp55441, ep456.comp54065 and ep456.comp52990 were changed by 1.9- to 3.9-fold. The target genes of miR166 and miR167, putative HD-ZIP transcription factor ep456.comp58119 and ARF-like unigenes, ep123.comp48918 and ep456.comp58489, respectively, also showed significant changes by 10- to 6.6-fold, indicating a regulatory relationship between the miRNA and the targets. Interestingly, we found that miR535 was notably enhanced in the mutant. MiR535 was expressed in a similar fashion to miR156 in the leaves and flowers of papaya and rice, and targeted the same *SPBL* genes [42]. Our result indicated a potentially redundant role for miR535 function in regulating *SPL* genes and in the multi-tepal mutant.

**Table 8:** Differentially expressed miRNAs and their target genes between the wild type and the mutant. Fold change = log2(expression value of each gene of the mutant/wild-type)

miRNA	Fold change	Target	Target fold change	Nr annotation
miR396	2.37	ep123.comp47283_c0_seq1	−2.11	ceramidase, putative [*Ricinuscommunis*]
		ep456.comp55669_c0_seq1	−2.60	LOC_Os02g47280.1|Oryza_sativa_subsp._japonica|GRF|LOC_Os02g47280.1
		ep456.comp58258_c0_seq1	2.47	ceramidase, putative [*Ricinuscommunis*]
		ep123.comp17494_c0_seq1	4.00	LOC_Os04g51190.3|Oryza_sativa_subsp._japonica|GRF|LOC_Os04g51190.3
		CL10167	4.28	LOC_Os06g02560.1|Oryza_sativa_subsp._japonica|GRF|LOC_Os06g02560.1
		ep456.comp59964_c0_seq3	8.22	LOC_Os04g51190.2|Oryza_sativa_subsp._japonica|GRF|LOC_Os04g51190.2
miR319	2.04	CL10849Contig1	−1.11	TPA: putative MYB DNA-binding domain superfamily protein [*Zea mays*]
		ep123.comp27081_c0_seq1	1.81	candidate developmental transcription factor TCP1 [*Phalaenopsis* hybrid cultivar]
		ep123.comp44934_c0_seq2	3.19	TCP family transcription factor [*Dendrobium* hybrid cultivar]
		ep123.comp31487_c0_seq1	4.09	candidate developmental transcription factor TCP1 [*Phalaenopsis* hybrid cultivar]
miR156	2.00	ep456.comp55441_c0_seq1	1.91	SQUAMOSA promoter-binding-like 3 [*Erycinapusilla*]
		ep456.comp59687_c2_seq1	2.26	MADS1 protein [*Eschscholziacalifornica*]
		ep456.comp54065_c0_seq1	2.36	SQUAMOSA promoter-binding-like 10 [*Erycinapusilla*]
		ep456.comp52990_c0_seq4	2.38	SQUAMOSA promoter-binding-like 7 [*Erycinapusilla*]
		ep456.comp58153_c0_seq3	2.45	MADS box protein [*Phalaenopsisequestris*]
		ep456.comp52990_c0_seq5	3.94	SQUAMOSA promoter-binding-like 9 [*Erycinapusilla*]
miR166	1.87	ep123.comp46514_c0_seq3	−4.20	PREDICTED: receptor-like serine/threonine-protein kinase SD1-8-like [*Vitisvinifera*]
		ep456.comp59626_c0_seq4	1.84	hypothetical protein SORBIDRAFT_01g019330 [*Sorghum bicolor*]
		ep456.comp56959_c0_seq2	2.55	Endosomal P24A protein precursor, putative [*Ricinuscommunis*]
		ep456.comp58119_c0_seq1	2.56	class III homeobox-leucine zipper protein [*Asparagus officinalis*]
		ep456.comp59626_c0_seq21	7.40	hypothetical protein SORBIDRAFT_01g019330 [*Sorghum bicolor*]
miR167	1.88	ep123.comp48918_c0_seq1	−10.23	PREDICTED: auxin response factor 2 [*Vitisvinifera*]
		ep456.comp58489_c0_seq4	6.67	PREDICTED: auxin response factor 2 [*Vitisvinifera*]
miR535	1.45	ep456.comp49040_c0_seq1	−5.89	PREDICTED: ELMO domain-containing protein A-like [*Glycine max*]
		ep456.comp40482_c0_seq1	−1.88	PREDICTED: putative pentatricopeptide repeat-containing protein [*Fragariavesca subsp.*vesca]
		ep456.comp57953_c0_seq2	10.40	S-adenosyl-L-methionine decarboxylase [*Dendrobiumcrumenatum*]
		ep456.comp52990_c0_seq4	2.38	SQUAMOSA promoter-binding-like 7 [*Erycinapusilla*]
miR398	−2.67	ep456.comp58639_c0_seq3	−7.82	hypothetical protein PRUPE_ppa020963mg [*Prunuspersica*]
		ep123.comp48546_c0_seq1	−3.23	PREDICTED: LOW QUALITY PROTEIN: DNA polymerase theta-like [*Vitisvinifera*]
		ep123.comp47734_c0_seq2	−2.29	PREDICTED: uncharacterized protein LOC100245378 [*Vitisvinifera*]
		ep456.comp58639_c0_seq4	2.00	hypothetical protein PRUPE_ppa020963mg [*Prunuspersica*]
		ep456.comp58639_c0_seq1	3.41	PREDICTED: LOW QUALITY PROTEIN: DNA polymerase theta-like [*Vitisvinifera*]
		ep123.comp44813_c0_seq1	1.14	hypothetical protein VITISV_023178 [*Vitisvinifera*]
		ep456.comp59280_c0_seq1	3.47	N-acetyltransferase, putative [*Ricinuscommunis*]
miR528	2.19	ep456.comp57151_c0_seq1	1.84	polyphenol oxidase [*Doritispulcherrima* x *Phalaenopsis*hybrid cultivar]
		ep456.comp53200_c0_seq2	2.87	TPA: hypothetical protein ZEAMMB73_977642 [*Zea mays*]
		ep456.comp217276_c0_seq1	5.34	glutaredoxin family protein [*Musa acuminata*]
		ep123.comp50029_c0_seq1	3.11	Endoribonuclease Dicer-like protein 4 [*Triticumurartu*]
miR6173	−1.11	ep123.comp48621_c0_seq3	−8.01	predicted protein [*Hordeumvulgaresubsp. vulgare*]
		ep123.comp41527_c0_seq11	−9.84	Mitochondrial protein, putative [*Medicago truncatula*]
		ep123.comp48621_c0_seq1	−8.01	TPA: hypothetical protein ZEAMMB73_631850 [*Zea mays*]
miR3630	1.96	ep123.comp42566_c0_seq1	10.71	SQUAMOSA promoter-binding-like 12 [*Erycinapusilla*]
miR5083	2.35	ep123.comp25253_c0_seq1	−3.60	Os03g0698500 [*Oryza sativa Japonica* Group]
		CL8873Contig1	−2.85	uncharacterized protein LOC100194037 [*Zea mays*]
		ep123.comp25253_c0_seq2	−3.60	Os03g0698500 [*Oryza sativa Japonica* Group]

Despite the conserved floral-related miRNAs, miR398 and miR528, which are majorly involved in plant stress responses, were also altered in the mutant. The former was 2.7-fold lower and the latter was about three-fold higher. These results indicated a potential link between the stress response and the mutant phenotype. Moreover, we noted that miR6173, miR3630 and miR5083 exhibited different specificities with changes ranging from one- to 2.4-fold, and their candidate target genes were also correspondingly changed by −9.8 to 10.7-fold (Table 8), indicating their potential role in floral patterning.

### Expression validation of miRNAs and their targets involved in multi-tepal development

**Fig. 9:**
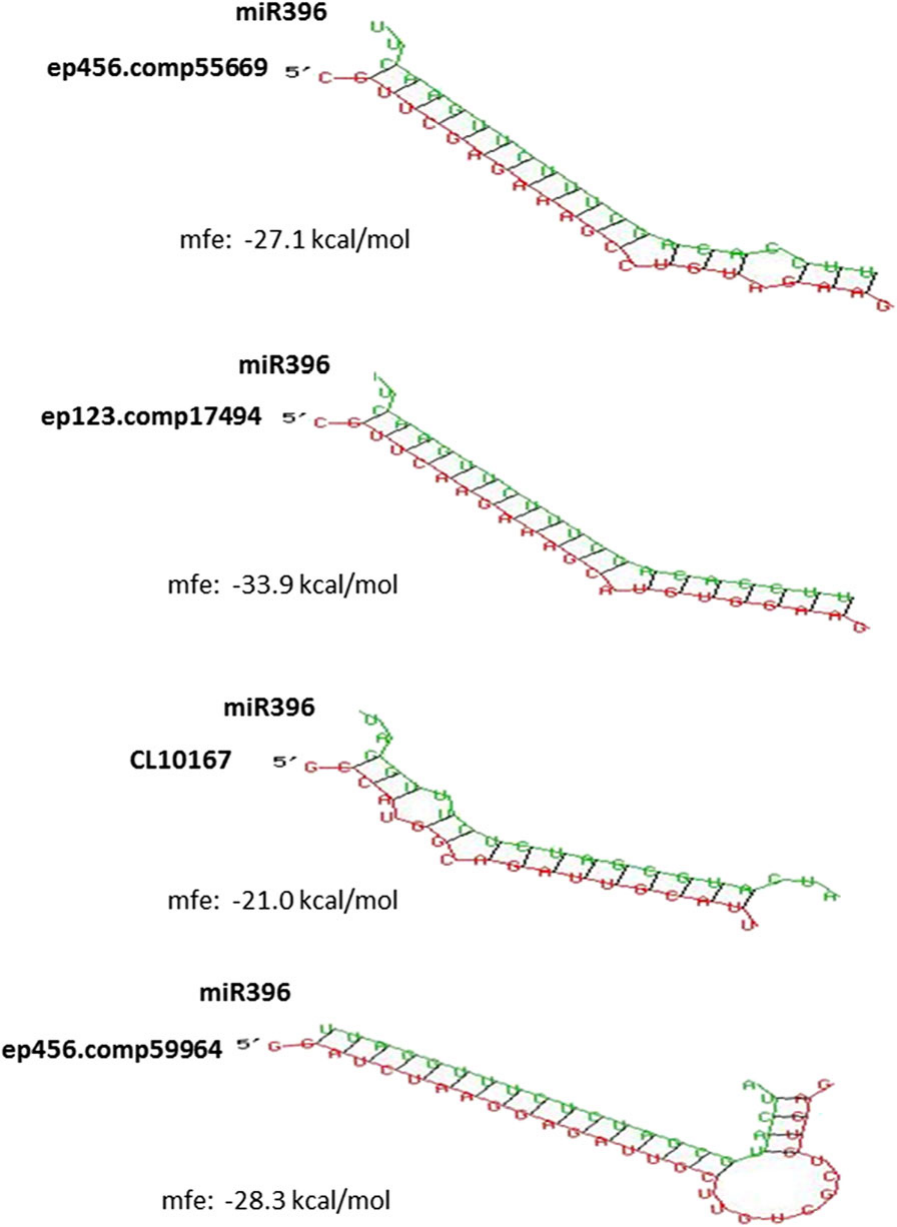
Comparison of complementarity profiles for miR396 with *GRF-like* genes. Free energies of the duplex structures were calculated using RNA hybrid software (http://bibiserv.techfak.uni-bielefeld.de/rnahybrid)

**Fig. 10:**
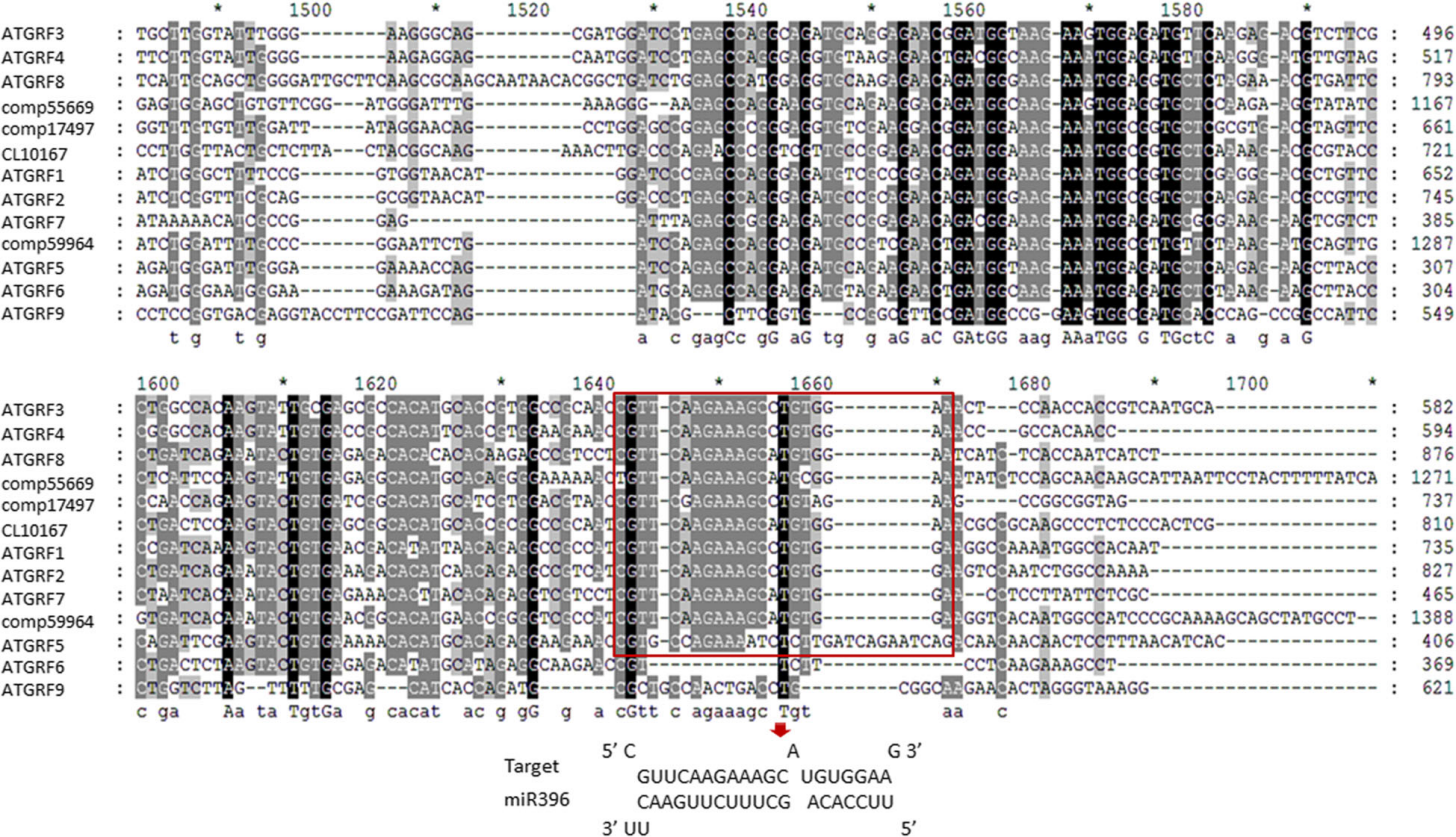
Sequence complementary to miR396 in four of the *GRF-like* genes. The comparison of complementary site of miR396 to *GRF-like* genes is indicated by the red frame

To validate the result obtained from small RNA sequencing and determine the potential roles of the miRNAs mentioned above, we confirmed the expressions of miR396 and miR319, and their correlated targets among different floral organs in the wild-type and mutant. Our previous studies in *Arabidopsis* and tobacco indicated that miR396 can regulate expression of *GRF* genes, which are known to be involved in the control of cell proliferation during leaf and flower development. miR396-overexpressing plants produced flowers with various deformations: fused petals, extremely bent pistils, unfused carpels and single carpels [43, 44]. In this work, four of the *GRF-like* genes were predicted to be the targets of miR396 in *C. goeringii. In silico* RNA hybridization revealed a sequence complementary to miR396 in these sequences, and the target position was similar to that in *Arabidopsis* (Figs. 9 and 10). Since the conserved regulation of *GRF* genes by miR396 has been broadly reported in both monocotyledons and dicotyledons, our findings indicated that *CgGRF-like* genes were putative targets of miR396 in *C. goeringii*.

**Fig. 11:**
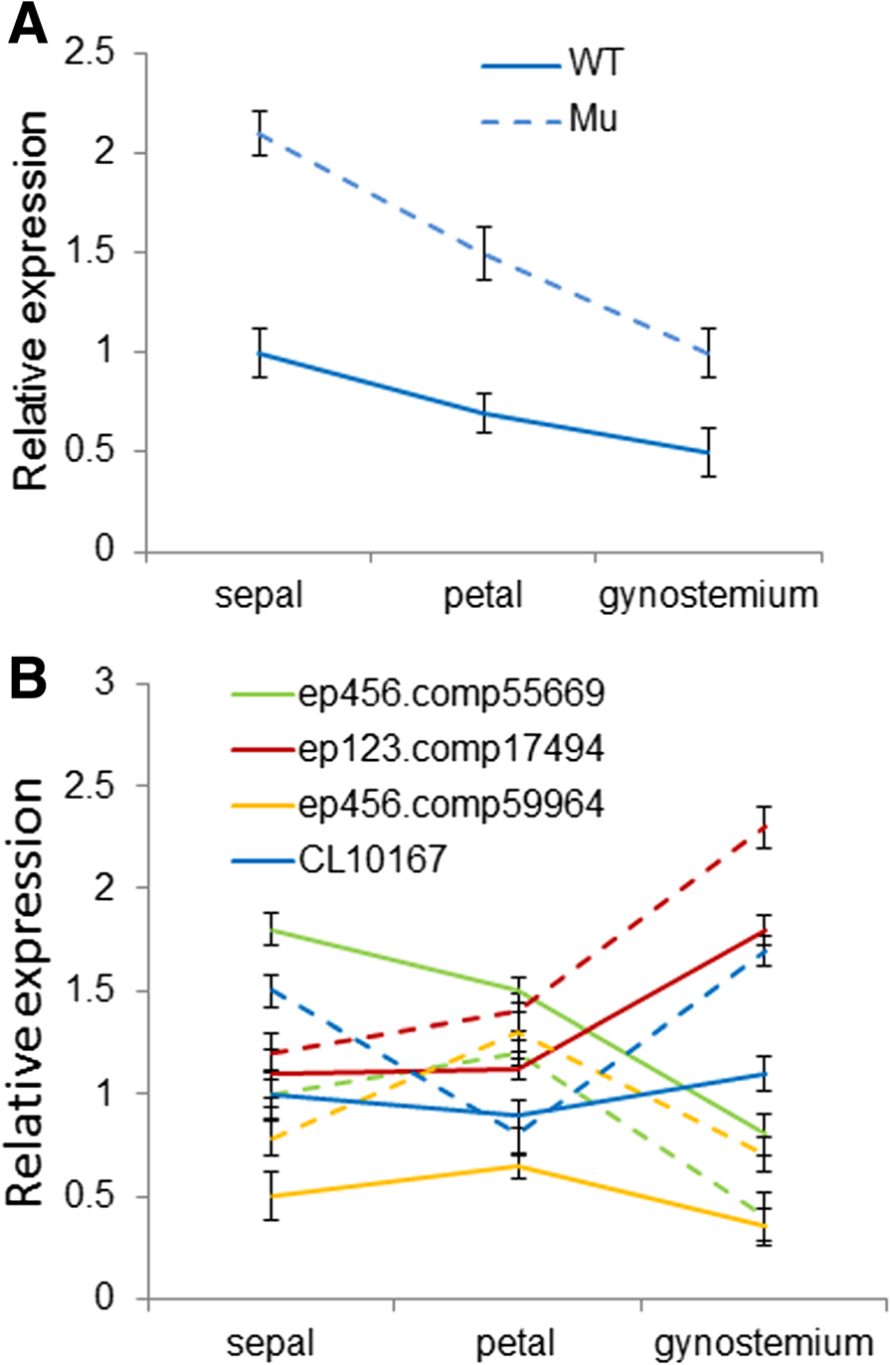
Transcript levels of miR396 (**a**) and *GRF-like* genes (**b**) in different floral organs of *C. goeringii* ‘Songmei’ (WT) and the multi-tepal mutant ‘Yuhudie’ (Mu) using a real-time RT-PCR assay. The ubiquitin gene served as the internal control. Error bars indicate the standard deviation of the mean (SD) (*n* = 3). Three replicates were analyzed, with similar results

Stem-loop real-time RT-PCR showed that the highest levels of miR396 were in sepals and then in petals and gynostemia in both wild-type and mutant plants, consistent with findings in *Arabidopsis.* However, expression was about two-fold higher in the mutant, especially in petals (Fig. 11a)*.* The levels of its putative targets, *GRF*-*like* genes, were also examined in different floral organs using real-time RT-PCR. Among them, expressions of the *GRF*-*like* transcripts ep123.comp17494 and ep456.comp55669 were up- and down-regulated in the mutant, respectively (red and green dotted lines, respectively, in Fig. 11b). The highest levels of ep123.comp17494 were in gynostemia in both the wild-type and mutant flowers, and were negatively correlated with levels of miR396. However, ep456.comp55669 was dramatically expressed in petals and sepals, similarly to miR396. In contrast, expression patterns of the *GRF-like* genes ep456.comp59964 and Cl10167 differed between the wild-type and the mutant. The former accumulated predominantly in the gynostemium and the latter in the petals in the mutant; however, no significant organ-specific enrichment was detected for either in the wild-type, and subsequently resulted in an overall decreased expression level compared to the mutant. These results followed similar trends to those of the read numbers and suggested an important role of 396/GRF pattern in the floral development of *C. goeringii.*

**Fig. 12:**
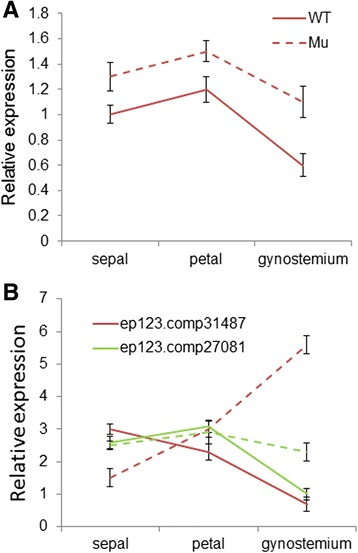
Transcript levels of miR319 (**a**) and TCP-like genes (**b**) in different floral organs of *C. goeringii* ‘Songmei’ (WT) and the multi-tepal mutant ‘Yuhudie’ (Mu) using a real-time RT-PCR assay. The *ubiquitin* gene served as the internal control. Error bars indicate the standard deviation of the mean (SD) (*n* = 3). Three replicates were analyzed, with similar results

Comparatively, expression of miR319 was also broadly detected in different organs, with higher abundance in the petals and lips. Consistent with the transcriptome deep sequencing, the overall expression level of miR319 was higher in the mutant than in the wild-type (Fig. 12a). In addition, the mRNA amounts of two *TCP-like* unigenes that showed sequence complementary to miR319 (Additional file 12) were also determined. Real-time PCR showed large increases in *TCP-like* gene ep123.comp31487 in the central part of the mutant flower, with a positive correlation with that of miR319 (Fig. 12b). There were no significant correlations between the expression levels of ep123.comp27081 and miR319. This could be due to non-cleavage repression, feedback regulation and spatial or temporal exclusion of miRNAs and their targets. The expression of other miRNAs leading to different levels of target repression, or other levels of regulation, such as promoter methylation and translational repression also exist. Our results showed that the relative abundance of these putative targeted sequences followed similar trends to those of the read numbers, and the same differential expression patterns were also observed between the wild-type and the mutant in technical replicates. This indicated that the comprehensive profiling of the *C. goeringii* miRNA provided a useful reference for further study. However, functional analysis is required to explore the regulatory mechanisms of various miRNAs and their targets contributing to the multi-tepal phenotype in the orchid.

## Discussion

### Transcriptome sequencing and the sequence annotation of *C. goeringii*

*C. goeringii* is a very important potted plant in China, and the multi-tepal mutant is popular in the commercial orchid market. However, little is known about the mechanisms responsible for floral patterning and multi-tepal development. Currently, next-generation sequencing technologies make it convenient to study the transcriptome of a particular organism or tissue to gain insight into biological processes, and numbers of these studies have been reported in many orchid plants. In *C. goeringii*, however, genomic information is currently unavailable. Our research primarily generated a large amount of cDNA sequences and miRNA data that will facilitate more detailed studies in *C. goeringii*, and will identify the genes controlling floral patterning in the multi-tepal mutant. In our study, a combination of wild-type and multi-tepal mutant libraries generated ~20 Gb of high quality reads that formed 98,446 unigenes, having an average sequence length of 989 bp. These unigenes were used in searches powered by the BLASTX algorithm and annotations against protein databases, like Nr, SwissProt, COG, GO and KEGG. In total, 78,175 sequences were identified through searches, and 20.6% of the unigenes had no homologs in the NCBI database.

**Fig. 13:**
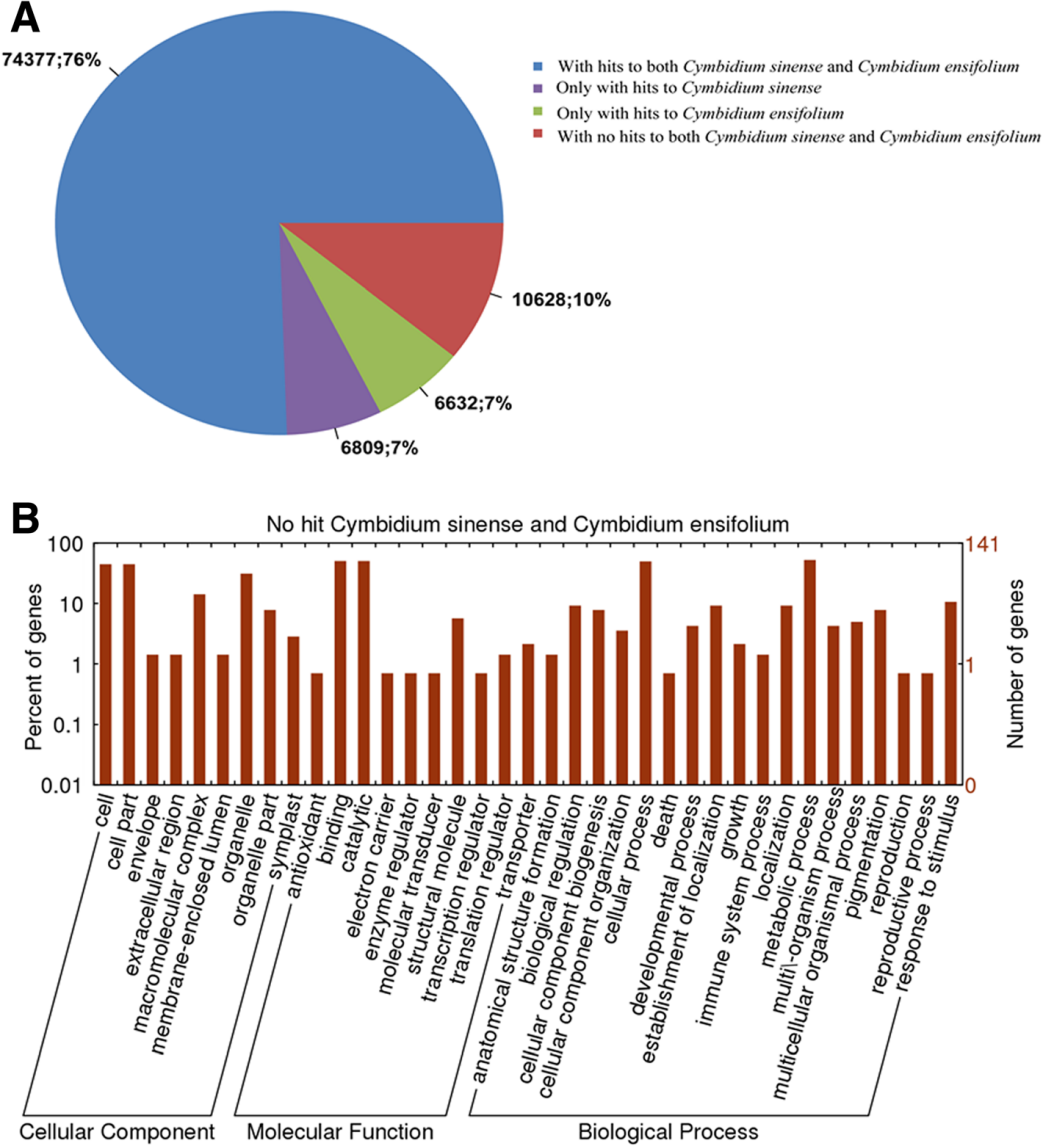
Comparison of *C. goeringii* floral transcriptome similarity with *C. sinense* and *C. ensifolium.* **a**: Similarity search of *C. goeringii* sequences against *C. ensifolium* and *C. sinense* sequences. **b**. Functional classification of unigenes unique to *C. goeringii*

When comparing with the orchid plants in other sub-families of the Orchidaceae, such as *P. equestris* and *D. officinale*, about 51% mapping rate was obtained, consistent with the similarity between *C. ensifolium* and *P. equestris* reported by Li et al. [45]. In contrast, comparison among three *Cymbidium* species, *C. ensifolium*, *C. sinense* and *C. goeringii*, showed almost 90% similarities. Among the aligned sequences, 74,377 (76%) unigenes had similarities with both *C. ensifolium* and *C. sinense*, whereas 10,628 (10%) isotigs had no similarity with either *C. ensifolium* or *C. sinense* (Fig. 13a). This may indicate that *C. goeringii* vegetative and reproductive growth contains many unique processes and pathways.

The GO analysis resulted in annotation of 4932 of the unigenes unique to *C. goeringii*, and the genes involved in metabolic processes, cellular processes and stimulus response were relatively more represented. For cellular components, the ‘cells’ and ‘cell part’ categories were most represented followed by ‘organelle’ (Fig. 13b); and for the molecular functions category, ‘binding’ and ‘catalytic activity’ were the most prevalent GO terms, followed by ‘structural molecular‘. All of these unique unigenes belonging to different GO terms may be important for traits specific to *C. goeringii*, such as special flower formation, scent production and environment adaptation, which represent a valuable resource for exploring the genetic diversity of *C. goeringii*, and for comparative genomic studies among orchid species.

### Floral homeotic genes related to multi-tepal development of *C. goeringii*

According to the floral quartet model, BC gene products and the SEPALATTA protein assembled into quaternary complexes, which specify the stamen, while C gene and *SEPALATTA* control carpel identity. AG proteins can form a homodimer and exert CArG-box binding activity. Orchids have unique morphologies, and previous studies in the orchid plant indicated a modified model of patterning, although most of the gene orthologs involved in the regulation of organ identity were highly conserved in evolution [46, 47]. For example, orchids typically have four *AP3*/*DEF*-*like* genes, representing the ancient gene Clades 1, 2, 3 and 4. High gene expression levels of Clades 1 and 2, and low levels of Clades 3 and 4, specify inner lateral tepals, whereas labellum development requires low levels of Clade 1 and 2 expression and high levels of Clade 3 and 4 expression [13, 14, 48]. Findings of duplicated four-clade B-class genes with differential expression patterns in orchid floral organs and their divergent protein behaviors support the unique evolutionary routes of B-class genes associated with unique floral organ development. However, orchids remain under-represented in molecular studies of floral development compared with other species-rich plant families.

In this study, we primarily identified many floral homeotic genes differentially expressed between the wild-type and mutant, which were probably related to multi-tepal formation, including homologs of the A-function gene *APETALA*, B-function gene *AP3*/*DEF*, and E-function genes *SEPALLATA/AGL.* More importantly, we found four *AG-like* C-class genes that were notably repressed in the mutant (Table 3). According to the floral quartet model, C-class genes specify stamen and carpel development and function in meristem determination because C-class mutants are indeterminate in whorl 4 and form a new C-class mutant flower instead of carpels. In core eudicots, C lineage MADS-box genes have been separated into euAG and PLENA lineages. After duplication, the primary C functions were subfunctionalized. For example, in *Antirrhinum majus*, PLENA has a main role in C functions, including the termination of floral meristem and the establishment of stamen and carpel identities, but the euAG sublineage gene FARINERRI contributes only to male fertility [49]. Duplications of C-class genes in monocots have revealed that their functions may diversify and become partially redundant. In maize, C-class *ZMM2* has a major function in organ identity, and *ZAG1* is predominant in floral meristem determinancy [50]. In rice, *OsMADS3* has a strong role in repressing lodicule development in whorl 2 and in specifying stamen identity, whereas *OsMADS58* contributes more to conferring floral meristem determinacy and regulating carpel development [51]. Orchid C-class genes were identified and contained duplication events. Previous study in *C. ensifolium* indicated that the major C function provided by *CeMADS1* and the paralogous *CeMADS2* may have functional redundancy in meristem activity [16]. Here, we found four unigenes that act as C-class *AG-like* genes and were significantly decreased in the mutant. A further analysis will reveal the potential roles and regulatory mechanisms of these paralogous genes.

### Other floral regulators related to multi-tepal development of *C. goeringii*

Along with the altered expression of ABCDE model genes, many other transcription factors that regulate class floral homeotic genes [52, 53], including the orthologs of *LUG* and *EMF2*, were up-regulated, whereas the expression of *CLF* orthologs were approximately five-fold lower in the mutant flower transcriptome. Other important categories of gene orthologs regulating patterning and symmetry also showed altered expression levels in the mutant floral transcriptome. For example, the putative orthologs of TCP and NAC. For the TCP ortholog, comparatively higher expression levels of four TCP-like genes were observed in the mutant transcriptome compared with the wild-type. Additionally, 19 of the putative NAC orthologs were also altered in the mutant floral transcriptome (Additional file 13).

There were also homologs of meristem activity regulators, such as *BAM* and *WUS*, which enhance meristem proliferation [54], found to have higher expression levels in the mutant floral transcriptome. In parallel, *GAI* and *ANT*, which are involved in decreasing meristem proliferation [55], also showed high levels in the mutant flower. Putative homologs of the genes *ER*, *ERL1* and *ERL2* encoding protein kinases that influence meristem cell fate and patterning in the inflorescence meristem [56], were also highly expressed. This *in silico* expression analysis of genes related to flower development and cell fate determination provided new insights into multi-tepal development.

### Integrated actions of multiple hormones involved in the multi-tepal development

During floral development, the floral organ identity genes specify the identity of different floral organs. However, to obtain their characteristic final size and shape, growth of the developing floral primordium needs to be tightly coordinated first through cell proliferation and then by cell expansion [9, 57]. Plant hormones play important roles in this progression, and the signaling pathways of phytohormones are interconnected in a complex network to regulate flower organ primordia formation, organ specification and final organ size [58].

In this study, transcriptome analyses showed that the plant hormone signal transduction pathway involving 70 unigenes was enriched with a *P*-value of 8.6E-21. Among them, auxin represents a master player as it acts not only as a local morphogenetic trigger in flower organ primordia formation, but also in concert with other hormones during further growth, patterning and reproductive organ development [59]. Interestingly, three well-known groups of early auxin-responsive genes—the *Aux/IAA*, *SAUR* and *GH3* gene families [33]—were generally repressed in the mutant. This repression of gene expression in the auxin signal pathway was well correlated with the retarded cell expansion and decreased cell size, which resulted in small and narrower tepals in the mutant.

Other hormones, such as cytokinin and gibberellin, are also critical for proper floral organ identity. Exogenous cytokinin applications and accumulation of endogenous cytokinin increase the flower number and induce an aberrant floral phenotype in several species. For example, an elevated level of endogenous cytokinin in *Arabidopsis* promotes cell division in the inflorescence meristem and the flower meristem, resulting in an increased number of flower organs [60]. In this study, the expressions of the ARR and AHP homologies in the cytokinin signaling pathway were also greatly up-regulated in the mutant, suggesting a positive regulation of cytokinin in the multi-tepal development. Gibberellin promotes the expression of floral homeotic genes, including *AP3*, *PI* and *AG* by antagonizing the effects of DELLA proteins—a family of nuclear growth repressors altering cell proliferation and expansion [37–39]. Our findings indicated that the expression of DELLA family genes and downstream responsive transcription factors were significantly altered, suggesting a critical role of gibberellin on floral patterning regulation. Moreover, gene homologies involved in abscisic acid, ethylene and brassinosteroid were also greatly altered in the mutant. These results suggest important roles for the hormone pathway in floral patterning regulation, and all of the candidate genes provide important clues to multi-tepal development.

### MiRNA and transcription factor networks regulating multi-tepal development

**Fig. 14:**
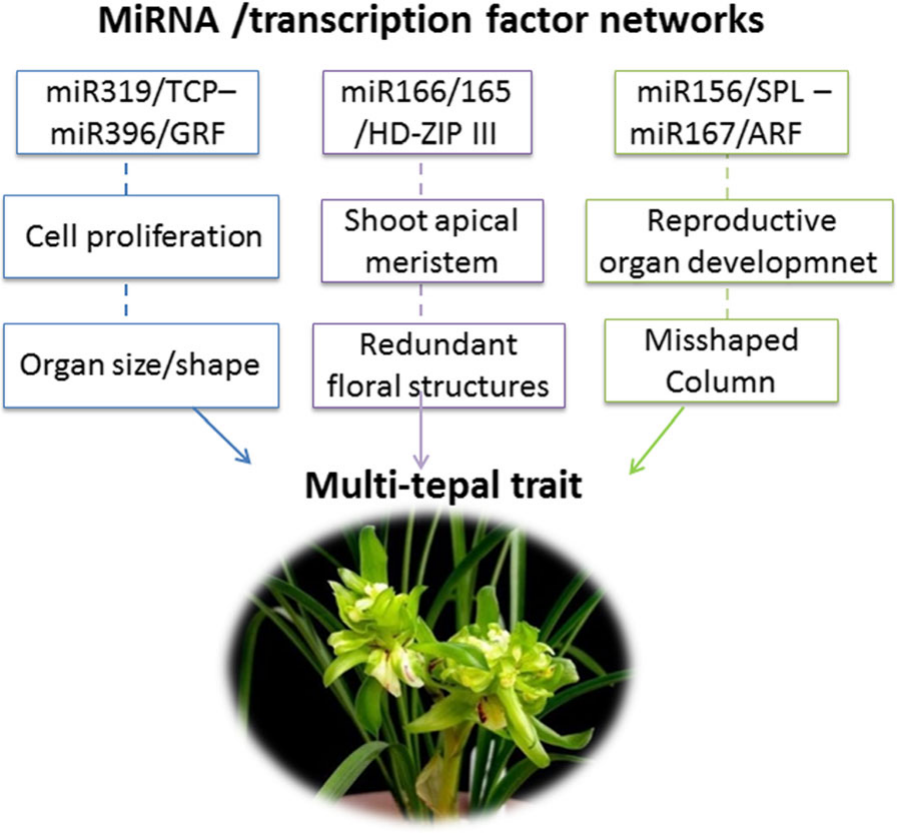
MiRNA/transcription factor networks contributing to the multi-tepal trait of *C. goeringii*

MiRNAs are not only regulators of gene expression, but also play an essential role in the coordination of complex developmental processes through their extensive integration within genetic networks. In our study, 11 out of 132 miRNA families exhibited fold changes of log_2_ > 1 and *P*-value < 0.05 between the wild-type and the mutant, functioning as putative regulators for multi-tepal development. These included the well-known floral-related miR156, miR166/165, miR167, miR319 and miR396 (Fig. 14).

MiR156 and miR167 are well known to regulate floral, especially reproductive structure, development through interference with the auxin signaling pathway. For example, miR156-targeted *SPL8* gene regulates gynoecium patterning in *Arabidopsis*, with down-regulation of miR156-targeted *SPL* genes resulting in a shortened style and an apically swollen ovary narrowing onto an elongated gynophore [61]. miR167 restricts *ARF6* and *ARF8* accumulation, resulting in failure to elongate filaments at anthesis (pre-anthesis filament elongation) and either lack of functional pollen grains or release of defective pollen grains before completion of filament elongation [62]. In this study, we found enhanced expression of miR156 and miR167, and corresponding alterations of their target transcripts in the *C. goeringii* multi-tepal mutant, which probably contributed to the significant deformation of its reproductive organs.

miR166/165 regulate *HD-ZIP III* transcripts through ARGONAUTE10 (AGO10) proteins to regulate shoot apical meristem formation and floral development by acting in parallel with the WUS–CLAVATA pathway [63]. In our work, homologs of these meristem activity regulators were induced in the mutant, and correlated well with the redundant floral structures of the multi-tepal mutant. However, further work is required to explore the targets and their regulatory mechanisms.

More importantly, miR396, another conserved miRNA, had the largest fold change difference between the wild-type and the mutant. The miR396 plays critical roles in plant leaf and flower development by modulating cell proliferation, at least to some extent. Seven of the nine members of the growth regulating factor family, *GRF1–4* and *GRF7–9*, are the targets of miR396 in *Arabidopsis. GRF*s together with the family of putative transcriptional co-activators encoded by the *GRF-INTERACTING FACTOR* (*GIF*) genes regulate organ size by maintaining cell proliferation, with mutants forming smaller and narrower leaves, whereas overexpression leads to larger leaf size. Plants expressing high levels of miR396 produce narrow leaves, fused tepals and abnormal productive organs, such as extremely bent pistils, unfused carpels and single carpels [43, 44, 64].

Interestingly, the expression of miR396 is itself regulated by *TCP4*, a member of the TEOSINTE BRANCHED1/CYCLOIDEA/PCF (TCP) transcription factor family. *TCP4* is a key regulator in petal development, to modulate cell division and cell differentiation, and is a target for miR319 [65]. Expression of a wild-type version of *TCP4* under the *AP3* promoter (AP3:TCP4) has very limited effect. By contrast, the AP3:mTCP4 lines with a miR319 resistant version exhibit a complete absence of petals and stamens. A point mutation in the miR319 target site of *TCP4* reduces the interaction with miR319 and causes higher levels of miR396 and lower amounts of *GRF* transcripts [66]. In this study, we found higher expressions of both miR319 and miR396, and corresponding changes of their target transcripts in the multi-tepal mutant. In light of the regulation of miR396 by *TCP4* in *Arabidopsis*, we hypothesize that a miR319/TCP4–miR396/GRF regulatory cascade also exists in *C. goeringii* to regulate cell proliferation. This probably contributed to the reduced cell size and narrow petals of the mutant.

## Conclusions

Orchid plants with multiple tepals have considerable ecological and cultural value. In this work, we analyzed a typical multi-tepal mutant of *C. goeringii*, which continued to produce sepals and petals instead of a gynostemium in the center of the flower, with misshapen lips and gynostemia. Aiming to reveal the networks involved in regulatory mechanisms of multi-tepal development, we compared the mRNA and small RNA profiles between the wild-type and the mutant. Comparative transcriptome analysis showed a great number of floral-related genes, as well as plant hormone-responsive genes contributing to multi-tepal development. Combined with miRNA sequencing data, two transcription factor/microRNA-based genetic pathways were considered to regulate multi-tepal development: well-known floral-related miR156/SPL and miR167/ARF regulatory modes probably contributing to reproductive organs development, and the miR319/TCP4–miR396/GRF regulatory cascade involved in cell proliferation in the multi-tepal development. Moreover, the comprehensive profiling of the *C. goeringii* transcriptome and miRNAome provided a useful reference for further investigations of miRNA in Orchidaceae.

## Methods

### Plant materials and growth conditions

Wild-type plants of *C. goeringii* ‘Songmei’ and the multi-tepal mutant ‘Yuhudie’ (the most widely known commercial cultivars in China) used in this study were artificially cultivated and collected from the cultivation base of Environmental Horticulture Research Institute, Guangdong Academy of Agricultural Sciences, China. All plants were grown and maintained in pots in a greenhouse at day/night with temperatures of 26/23 °C and photoperiod of 16/8 h.

### Library construction and Illumina sequencing

The primary aim of this study was to identify the genes responsible for the multi-tepal phenotype, including floral trait mutations such as multi-sepals and -petals, misshapen labella and gynostemium–petal fusions. We therefore respectively extracted the total RNA from the sepal, petal, labellum and gynostemium, rather than from the whole flower, to compare the unigene sets corresponding to individual floral organs. The cDNA and small RNA libraries were prepared from an equal mixture of RNAs isolated from different floral organs as outlined above. Each tissue sample consisted of individual floral organs isolated from 5–8 natural flowers. The mRNAs were purified from total RNA using the Oligotex mRNA Midi Kit (QIAGEN, Germany) and quantified using a Nano-Drop 2000 spectrophotometer (Thermo Scientific, USA) to generate the cDNA library according to the Illumina manufacturer’s instructions as described in our previous work [18]. Briefly, mRNAs were isolated from Total RNA and fragmented to approximately 200 bp. Subsequently, the collected mRNAs were subjected to first strand and second strand cDNA synthesis following by adaptor ligation and enrichment with a low-cycle according to instructions of TruSeq® RNA HT Sample Prep Kit (Illumina, USA). The purified library products were evaluated using the Agilent 2200 TapeStation and Qubit®2.0(Life Technologies,USA) and then diluted to 10 pM for cluster generation in situ on the HiSeq2500 pair-end flow cell followed by sequencing (2 × 100 bp) on HiSeq 2500. For small RNA sequencing, a total amount of 3 μg of total RNA from the wild-type and mutant were used as input material to construct small RNA libraries, following the procedures described by Li et al. [28]. Briefly, RNAs were ligated with 3’RNA adapter, and followed by 5’adapter ligation. Subsequently, the adapter-ligated RNAs were subjected to RT-PCR and amplified with a low-cycle. Then the PCR products were size selected by PAGE gel according to instructions of TruSeq® Small RNA Sample Prep Kit (Illumina, USA). The purified library products were evaluated using the Agilent 2200 TapeStation and diluted to 10 pM for cluster generation in situ on the HiSeq2500 single-end flow cell followed by sequencing (1 × 50 bp) on HiSeq 2500.

### Sequence assembly and annotation

The resulting raw sequence reads with weak signal or low quality were screened and trimmed by GS FLX pyrosequencing software, to yield a final dataset comprising high-quality (HQ) (>99.5% accuracy of single-base reads) sequences. Prior to assembly, primer and adapter sequences were trimmed from the HQ dataset, and sequences shorter than 50 bp were removed. The remaining data were assembled into unique sequences (including contigs) using trinityrn (http://trinityrnaseq.sourceforge.net/analysis/extract_proteins_from_trinity_transcripts.html).

For unigene annotations, the genes in which protein-coding sequences were found were searched against the Nr database (http://www.ncbi.nlm.nih.gov) and the SwissProt protein database (http://www.expasy.ch/sprot) using the BLASTP algorithm with an E-value cut-off of 10^−5^. The remaining sequences were searched against public databases, using the BLASTX algorithm with an E-value cut-off of 10^−5^.

GO classification was obtained by GO terms in the database (http://www.geneontology.org/). The unigene sequences were also aligned to the COG database to predict and classify functions. For identifying the transcription factors of *C. goeringii*, the protein sequence set of each transcription factor family from rice were downloaded from the PTFDB (http://planttfdb.cbi.pku.edu.cn/) and BLASTed against the sequences in our dataset with use of the TBLASTN programs. Sequence similarity was considered significant at E-value < 10^−5^. Pathway assignments were made according to the KEGG mapping project (http://www.genome.jp/). Enzyme commission (EC) numbers were assigned to unique sequences that had BLASTX scores with an E-value cut-off of 10^−5^ as determined by searching the KEGG database. The unique sequences were allocated to specific biochemical pathways according to the corresponding EC distribution in the KEGG database.

### DEG analysis

The gene expression level was calculated by the FPKM value: FPKM = [total transcript fragments/mapped fragments (millions)] × transcript length (kb). The significance of difference in gene expression between the wild-type and mutant was determined using edgeR. False discovery rate (FDR) was applied to identify the threshold of the P-value in multiple tests; with FDR < 0.05 and |log_2_ ratio| > 1 (two-fold change) as the threshold for significant difference in gene expression.

The DEGs were annotated using GO and KEGG enrichment analyses according to a method similar to that described by Wang, which firstly mapped all DEGs to GO terms (or KEGG pathways) in the database (http://www.geneontology.org/, http://www.genome.ad.jp/) and calculated gene numbers for every term (or pathway). This was followed by a hypergeometric test to find the significantly enriched terms in DEGs compared with the genome background. We used the corrected *P*-value ≤ 0.05 or Q-value ≤ 0.05 as a threshold for the significantly enriched GO terms or KEGG pathways, respectively, in DEGs.

### Bioinformatic analysis of small RNA deep-sequencing data

The small RNA data output from Illumina was filtered and the sequence mapping reads with poly-N, 5′-adapter contaminants, low quality or lacking the 3′-adapter or inset tags were removed. The clean data with read lengths in a specific size range of 17–30 nt were chosen for further analysis. These small RNA reads were mapped to RepeatMasker (www.repeatmasker.org), Rfam (rfam.janelia.org), UCSC (gtrnadb.ucsc.edu) and NONCODE (www.noncode.org/NONCODERv3/download.html), to distinguish miRNAs from the tags originating from protein-coding genes, repeat sequences, rRNA, tRNA, snRNA, snoRNA and piRNA.

### Conserved miRNA alignment and its expression

All unique reads left from the previous screening were searched against the miRBase (version: 21.0) using BLASTN with the same parameters values as used in the miRBase Web-Blast server (−w 4 –r 5 –q −4). Unique reads that were identical to or had less than three mismatches to known miRNAs were regarded as potential conserved miRNAs in *C. goeringii*. The counts of unique reads were normalized to reads per million (RPM) by dividing the raw read count by the total number of reads in each library. The expression profiles for each miRNA family were calculated by summing all reads annotated to the same miRNA family in each library. MiRNAs that had change ratios of > 2 or < 0.5 (fold change log_2_ > 1 or < −1) and *P* < 0.05 were set as the threshold for significant differential expression by default. Statistical analysis was performed using the method described by Audic and Claverie.

### Novel miRNA prediction

The miRNA precursor is characterized by its hairpin structure, which can be used to predict novel miRNA. Here, we used the software Mireap (https://sourceforge.net/projects/mireap/) to predict novel miRNA through exploring the secondary structure by identifying the Dicer cleavage site and the minimum free energy of the small RNA tags unannotated in the previous steps. The parameter value –f: (Flank sequence length of miRNA precursor) = 100 and the free energy < −20 kcal/mol.

### Target gene prediction

Two computational approaches were applied to predict miRNA target transcripts in this study as described by Chao et al. (2014). In the first approach, miRNAs were searched against our EST dataset with -g (gapped alignment) F (false) appended for BLASTN to prevent the insertion of gaps in the middle of an alignment. Alignments containing positions 2–12 of the miRNA with a mapping length over 16 nt and ≤ 3 mismatches were considered to be miRNA target candidates. In the second procedure, genomic data of *Arabidopsis* were downloaded from the TAIR10 database (http://www.arabidopsis.org/index.jsp) and BLASTed against our EST dataset of *C. goeringii* using a cut-off value of 1e-30. An EST was considered a candidate target gene if its best BLAST match was a target of *Arabidopsis* miRNAs listed in the *Arabidopsis* small RNA project (ASRP) database (http://asrp.cgrb.oregonstate.edu/) [67].

### Stem-loop RT-PCR of miRNAs and RT-qPCR of target genes

Individual floral organs of wild-type and multi-tepal mutant were used, independently, to extract RNA. The cDNA of the mature miRNA was prepared using the miRNA reverse transcription kit M-MLV (Takara, China), and the reverse-transcribed products were used as the template for RT-qPCR with gene-specific primers. The U6 snRNA was used for normalization. The miRNA specific stem-loop primers and gene-specific RT-qPCR primers were designed according to the rules described in Chen et al. [68]. For the RT-qPCR of target genes, total RNA extracted from different tissue types were reverse-transcribed by oligo(dT) primed cDNA synthesis protocol (Fermentas). The resulting cDNA was subjected to relative quantitative PCR using Bio-Rad CFX-96 RealTime PCR System (Bio-Rad, USA) in a final volume of 20 μl containing 2 μl of cDNA and 10 μl of SYBR premix Ex-taq™ (Takara, Japan). Ubiquitin was used as an internal control for normalization to make a comparison of gene expression level between the accessions. For each reported result at least three independent biological samples were subjected to a minimum of three technical replicates. The primers designed with Primer 5.0 software are listed in Additional file 14.

## Additional files

Additional file 1: Paradermal view of the epidermal cells of the petal both in the wild type and the mutant. (TIF 1951 kb)

Additional file 2: The length distribution of assembled unigenes. The x-axis represents the sequence length in base pairs. The y-axis represents the unigenes number. (TIF 478 kb)

Additional file 3: Details of the pathway annotations for the unigene sets by KEGG (XLS 1399 kb)

Additional file 4: Gene ID and transcript fold changes of the DEGs (differentially expressed genes) between the wild type and mutant (XLS 2888 kb)

Additional file 5: DEG-enrichment analysis by KEGG metabolic pathway classification. (XLS 619 kb)

Additional file 6: A total of 110 MADS-box genes in *C. goeringii*. (XLS 163 kb)

Additional file 7: Gene ID and fold changes of the unigenes involved in plant hormone signal transduction pathway annotated by KEGG pathway. (XLS 42 kb)

Additional file 8: Length distribution of small RNAs derived from Cymbidium *goeringii* ‘Songmei’ (WT) and the multi-tepal mutant ‘Yuhudie’ (Mu). (TIF 1182 kb)

Additional file 9: Distribution of the conserved miRNA families in Cymbidium *goeringii* ‘Songmei’ (WT) and the multi-tepal mutant ‘Yuhudie’ (Mu). (XLS 46 kb)

Additional file 10: putative target genes of the conserved miRNAs predicted in this study. (XLS 403 kb)

Additional file 11 Predicted miRNA target genes involved in floral development/flowering time. (XLS 79 kb)

Additional file 12: Phylogenetic analysis of *Cymbidium goeringii TCP–like* genes with their homologues in *Arabidopsis* and the sequence complementary to miR396 using RNA hybrid software (http://bibiserv.techfak.uni-bielefeld.de/rnahybrid) (TIF 496 kb)

Additional file 13: Gene ID and fold changes of the unigenes regulating floral homeotic genes and floral patterning (XLS 43 kb)

Additional file 14: The primers of stem-loop RT-PCR and qRT-PCR. (DOC 37 kb)

## Abbreviations

AG: AGAMOUS
AHP: Histidine phosphotransfer protein
AP1: APETALA1
AP2/ERF: APETALA2/Ethylene Responsive Factor
AP3: APETALA3
ARF: Auxin response factor
ARR: Response regulator
Aux/IAA: Auxin/indole-3-acetic acid
BAM: Barely Any Meristem
bHLH: basic helix-loop-helix
bZIP: Basic leucine zipper
CER: Cytokinin receptor
CLF: Curly Leaf
COG: Clusters of Orthologous Groups
DEF: DEFICIENS
DEGs: Differentially expressed genes
EMF2: Embryonic Flowers
FAR: Far-red impaired Response
GAI: GA insensitive
GH3: Auxin-responsive Gretchen Hagen3
GIF: GRF-interaction Factor
GO: Gene ontology
GRF: Growth-regulating factor
HD-ZIP: Homeobox-leucine zipper factors
KEGG: Kyoto encyclopedia of genes and genomes
LUG: Leunig
MYB: Myeloblastosis transcription factors
NAC: NAM, ATAF, and CUC transcription factor
NF-Y: Nuclear transcription factor
NIN: Nodule inception
RGL: Repressor of GA-Like
RPM: Reads per million
SAM: Shoot apical meristem
SAUR: Small auxin up RNA
SPL: SQUAMOSA promoter binding protein-like
TALE: Three amino acid loop extension family
TCP: TEOSINTE BRANCHED1/CYCLOIDEA/PC transcription factors
WUS: Wuschel.

## References

[1] Du Puy DJ, Cribb P, Tibbs M (2007). The Genus *Cymbidium*.

[2] Wu ZY, Raven PH, Hong DY (2009). Flora of China.

[3] Chung MY, Chung MG (2003). The breeding systems of *Cremastra appendiculata* and *Cymbidium goeringii*: high levels of annual fruit failure in two self-compatible orchids. Ann Bot Fenn.

[4] Rajkumari JD, Longjam RS. Orchid flower evolution. J Genet. 2005;84(1):81–4. 10.1007/BF02715895 15876589

[5] Yukawa T, Stern WL (2002). Comparative vegetative anatomy and systematics of *Cymbidium* (Cymbidieae : Orchidaceae). Bot J Linn Soc.

[6] Ning H, Zhang C, Fu J, Fan Y. Comparative transcriptome analysis of differentially expressed genes between the curly and normal leaves of Cymbidium goeringii var. longibracteatum. Genes & Genomics. 2016;38(10):985–98.

[7] Parcy F, Nilsson O, Busch MA, Lee I, Weigel D (1998). A genetic framework for floral patterning. Nature.

[8] Lohmann JU, Hong RL, Hobe M, Busch MA, Parcy F, Simon R, Weigel D (2001). A molecular link between stem cell regulation and floral patterning in *Arabidopsis*. Cell.

[9] Krizek BA, Fletcher JC (2005). Molecular mechanisms of flower development: An armchair guide. Nat Rev Genet.

[10] Bowman JL, Smyth DR, Meyerowitz EM (2012). The ABC model of flower development: then and now. Development.

[11] Coen ES, Meyerowitz EM (1991). The War of the Whorls - Genetic Interactions Controlling Flower Development. Nature.

[12] Favaro R, Pinyopich A, Battaglia R, Kooiker M, Borghi L, Ditta G, Yanofsky MF, Kater MM, Colombo L (2003). MADS-box protein complexes control carpel and ovule development in *Arabidopsis*. Plant Cell.

[13] Mondragon-Palomino M, Theissen G (2009). Why are orchid flowers so diverse? Reduction of evolutionary constraints by paralogues of class B floral homeotic genes. Ann Bot-London.

[14] Mondragon-Palomino M, Theissen G (2011). Conserved differential expression of paralogous DEFICIENS- and GLOBOSA-like MADS-box genes in the flowers of Orchidaceae: refining the 'orchid code'. Plant J.

[15] Hsu HF, Hsu WH, Lee YI, Mao WT, Yang JY, Li JY, Yang CH. Model for perianth formation in orchids. Nat Plants. 2015;1(5):15046.

[16] Wang SY, Lee PF, Lee YI, Hsiao YY, Chen YY, Pan ZJ, Liu ZJ, Tsai WC (2011). Duplicated C-Class MADS-Box Genes Reveal Distinct Roles in Gynostemium Development in *Cymbidium ensifolium* (Orchidaceae). Plant Cell Physiol.

[17] Albert VA, Carretero-Paulet L (2015). A genome to unveil the mysteries of orchids. Nat Genet.

[18] Yang FX, Zhu GF. Digital gene expression analysis based on de Novo transcriptome assembly reveals new genes associated with floral organ differentiation of the orchid plant cymbidium ensifolium. Plos One. 2015;10(11):e0142434. 10.1371/journal.pone.0142434 PMC4651537 26580566

[19] Zhang JX, Wu KL, Zeng SJ, da Silva JAT, Zhao XL, Tian CE, Xia HQ, Duan J. Transcriptome analysis of Cymbidium sinense and its application to the identification of genes associated with floral development. Bmc Genomics. 2013;14(1):279. 10.1186/1471-2164-14-279 PMC3639151 23617896

[20] Bartel DP (2004). MicroRNAs: Genomics, biogenesis, mechanism, and function. Cell.

[21] Mallory AC, Vaucheret H (2006). Functions of microRNAs and related small RNAs in plants. Nat Genet.

[22] Kurihara Y, Watanabe Y (2004). *Arabidopsis* micro-RNA biogenesis through Dicer-like 1 protein functions. P Natl Acad Sci USA.

[23] Brodersen P, Sakvarelidze-Achard L, Bruun-Rasmussen M, Dunoyer P, Yamamoto YY, Sieburth L, Voinnet O (2008). Widespread translational inhibition by plant miRNAs and siRNAs. Science.

[24] Lin CS, Chen JJW, Huang YT, Hsu CT, Lu HC, Chou ML, Chen LC, Ou CI, Liao DC, Yeh YY (2013). Catalog of *Erycina pusilla* miRNA and categorization of reproductive phase-related miRNAs and their target gene families. Plant Mol Biol.

[25] An FM, Hsiao SR, Chan MT. Sequencing-Based Approaches Reveal Low Ambient Temperature-Responsive and Tissue-Specific MicroRNAs in Phalaenopsis Orchid. Plos One. 2011;6(5):e18937. 10.1371/journal.pone.0018937 PMC3089612 21573107

[26] Chao YT, Su CL, Jean WH, Chen WC, Chang YCA, Shih MC (2014). Identification and characterization of the microRNA transcriptome of a moth orchid *Phalaenopsis aphrodite*. Plant Mol Biol.

[27] Meng YJ, Yu DL, Xue J, Lu JJ, Feng SG, Shen CJ, Wang HZ. A transcriptome-wide, organ-specific regulatory map of Dendrobium officinale, an important traditional Chinese orchid herb. Sci Rep-UK. 2016;6:18864. 10.1038/srep18864 PMC4702150 26732614

[28] Li XB, Jin F, Jin L, Jackson A, Ma X, Shu XL, Wu DX, Jin GQ. Characterization and comparative profiling of the small RNA transcriptomes in two phases of flowering in Cymbidium ensifolium. Bmc Genomics. 2015;16(1):622. 10.1186/s12864-015-1764-1 PMC4546042 26289943

[29] Mizukami Y, Fischer RL (2000). Plant organ size control: AINTEGUMENTA regulates growth and cell numbers during organogenesis. P Natl Acad Sci USA.

[30] Drews GN, Bowman JL, Meyerowitz EM (1991). Negative Regulation of the *Arabidopsis* Homeotic Gene Agamous by the Apetala2 Product. Cell.

[31] Kumimoto RW, Zhang Y, Siefers N, Holt BF (2010). NF-YC3, NF-YC4 and NF-YC9 are required for CONSTANS-mediated, photoperiod-dependent flowering in *Arabidopsis thaliana*. Plant J.

[32] Kumaran MK, Bowman JL, Sundaresan V (2002). YABBY polarity genes mediate the repression of KNOX homeobox genes in *Arabidopsis*. Plant Cell.

[33] Guilfoyle TJ, Hagen G (2007). Auxin response factors. Curr Opin Plant Biol.

[34] Inoue T, Higuchi M, Hashimoto Y, Seki M, Kobayashi M, Kato T, Tabata S, Shinozaki K, Kakimoto T (2001). Identification of CRE1 as a cytokinin receptor from *Arabidopsis*. Nature.

[35] Hwang I, Sheen J (2001). Two-component circuitry in *Arabidopsis* cytokinin signal transduction. Nature.

[36] Mason MG, Mathews DE, Argyros DA, Maxwell BB, Kieber JJ, Alonso JM, Ecker JR, Schaller GE (2005). Multiple type-B response regulators mediate cytokinin signal transduction in *Arabidopsis*. Plant Cell.

[37] Achard P, Vriezen WH, Van Der Straeten D, Harberd NP (2003). Ethylene regulates *Arabidopsis* development via the modulation of DELLA protein growth repressor function. Plant Cell.

[38] King KE, Moritz T, Harberd NP (2001). Gibberellins Are Not Required for Normal Stem Growth in *Arabidopsis thaliana* in the Absence of GAI and RGA. Genetics.

[39] Yamaguchi N, Winter CM, Wu MF, Kanno Y, Yamaguchi A, Seo M, Wagner D (2014). Gibberellin Acts Positively Then Negatively to Control Onset of Flower Formation in *Arabidopsis*. Science.

[40] Park SY, Fung P, Nishimura N, Jensen DR, Fujii H, Zhao Y, Lumba S, Santiago J, Rodrigues A, Chow TFF (2009). Abscisic Acid Inhibits Type 2C Protein Phosphatases via the PYR/PYL Family of START Proteins. Science.

[41] Ma Y, Szostkiewicz I, Korte A, Moes D, Yang Y, Christmann A, Grill E (2009). Regulators of PP2C Phosphatase Activity Function as Abscisic Acid Sensors. Science.

[42] Liang G, Li Y, He H, Wang F, Yu DQ (2013). Identification of miRNAs and miRNA-mediated regulatory pathways in *Carica papaya*. Planta.

[43] Liang G, He H, Li Y, Wang F, Yu DQ (2014). Molecular Mechanism of microRNA396 Mediating Pistil Development in *Arabidopsis*. Plant Physiol.

[44] Yang FX, Liang G, Liu DM, Yu DQ (2009). *Arabidopsis* MiR396 Mediates the Development of Leaves and Flowers in Transgenic Tobacco. J Plant Biol.

[45] Li XB, Luo J, Yan TL, Xiang L, Jin F, Qin DH, Sun CB, Xie M. Deep sequencing-based analysis of the cymbidium ensifolium floral transcriptome. Plos One. 2013;8(12):e85480. 10.1371/journal.pone.0085480 PMC3877369 24392013

[46] Aceto S, Gaudio L (2011). The MADS and the Beauty: Genes Involved in the Development of Orchid Flowers. Curr Genomics.

[47] Tsai WC, Hsiao YY, Pan ZJ, Chen HH (2010). Molecular Mechanisms Underlying Orchid Floral Morphogenesis. Acta Hortic.

[48] Chang YY, Kao NH, Li JY, Hsu WH, Liang YL, Wu JW, Yang CH (2010). Characterization of the Possible Roles for B Class MADS Box Genes in Regulation of Perianth Formation in Orchid. Plant Physiol.

[49] Davies B, Motte P, Keck E, Saedler H, Sommer H, Schwarz-Sommer Z (1999). PLENA and FARINELLI: redundancy and regulatory interactions between two *Antirrhinum* MADS-box factors controlling flower development. Embo J.

[50] Mena M, Ambrose BA, Meeley RB, Briggs SP, Yanofsky MF, Schmidt RJ (1996). Diversification of C-function activity in maize flower development. Science.

[51] Yamaguchi T, Lee DY, Miyao A, Hirochika H, An GH, Hirano HY (2006). Functional diversification of the two C-class MADS box genes *OSMADS3* and *OSMADS58* in *Oryza sativa*. Plant Cell.

[52] Lohmann JU, Weigel D (2002). Building beauty: The genetic control of floral patterning. Dev Cell.

[53] Yruela I (2015). Plant development regulation: Overview and perspectives. J Plant Physiol.

[54] Leibfried A, To JPC, Busch W, Stehling S, Kehle A, Demar M, Kieber JJ, Lohmann JU (2005). WUSCHEL controls meristem function by direct regulation of cytokinin-inducible response regulators. Nature.

[55] Gonzalez N, Vanhaeren H, Inze D (2012). Leaf size control: complex coordination of cell division and expansion. Trends Plant Sci.

[56] Mandel T, Moreau F, Kutsher Y, Fletcher JC, Carles CC, Williams LE (2014). The ERECTA receptor kinase regulates *Arabidopsis* shoot apical meristem size, phyllotaxy and floral meristem identity. Development.

[57] Zik M, Irish VF (2003). Flower development: Initiation, differentiation, and diversification. Annu Rev Cell Dev Bi.

[58] Davies PJ, Davies PJ (1995). The Plant Hormones: Their Nature, Occurrence, and Functions. Plant Hormones: Physiology, Biochemistry and Molecular Biology.

[59] Strader LC, Zhao Y (2016). Auxin perception and downstream events. Curr Opin Plant Biol.

[60] Bartrina I, Otto E, Strnad M, Werner T, Schmulling T (2011). Cytokinin Regulates the Activity of Reproductive Meristems, Flower Organ Size, Ovule Formation, and Thus Seed Yield in *Arabidopsis thaliana*. Plant Cell.

[61] Wang JW, Czech B, Weigel D (2009). miR156-Regulated SPL Transcription Factors Define an Endogenous Flowering Pathway in *Arabidopsis thaliana*. Cell.

[62] Wu MF, Tian Q, Reed JW (2006). *Arabidopsis* microRNA167 controls patterns of ARF6 and ARF8 expression, and regulates both female and male reproduction. Development.

[63] Kim J, Jung JH, Reyes JL, Kim YS, Kim SY, Chung KS, Kim JA, Lee M, Lee Y, Kim VN (2005). microRNA-directed cleavage of ATHB15 mRNA regulates vascular development in *Arabidopsis* inflorescence stems. Plant J.

[64] Rodriguez RE, Mecchia MA, Debernardi JM, Schommer C, Weigel D, Palatnik JF (2010). Control of cell proliferation in *Arabidopsis thaliana* by microRNA miR396. Development.

[65] Nag A, King S, Jack T (2009). miR319a targeting of TCP4 is critical for petal growth and development in *Arabidopsis*. P Natl Acad Sci USA.

[66] Schommer C, Debernardi JM, Bresso EG, Rodriguez RE, Palatnik JF (2014). Repression of Cell Proliferation by miR319-Regulated TCP4. Mol Plant.

[67] Allen E, Xie ZX, Gustafson AM, Carrington JC (2005). microRNA-directed phasing during trans-acting siRNA biogenesis in plants. Cell.

[68] Chen CF, Ridzon DA, Broomer AJ, Zhou ZH, Lee DH, Nguyen JT, Barbisin M, Xu NL, Mahuvakar VR, Andersen MR et al. Real-time quantification of microRNAs by stem-loop RT-PCR. Nucleic Acids Res. 2005;33(20):e179. 10.1093/nar/gni178 PMC1292995 16314309

