# Supplementary figures and images for: Integrated mRNA and microRNA transcriptome variations in the multi-tepal mutant provide insights into the floral patterning of the orchid Cymbidium goeringii
Source: BMC Genomics. 2017 May 11;18:367. doi: 10.1186/s12864-017-3756-9 (PMC5426072; doi:10.1186/s12864-017-3756-9)

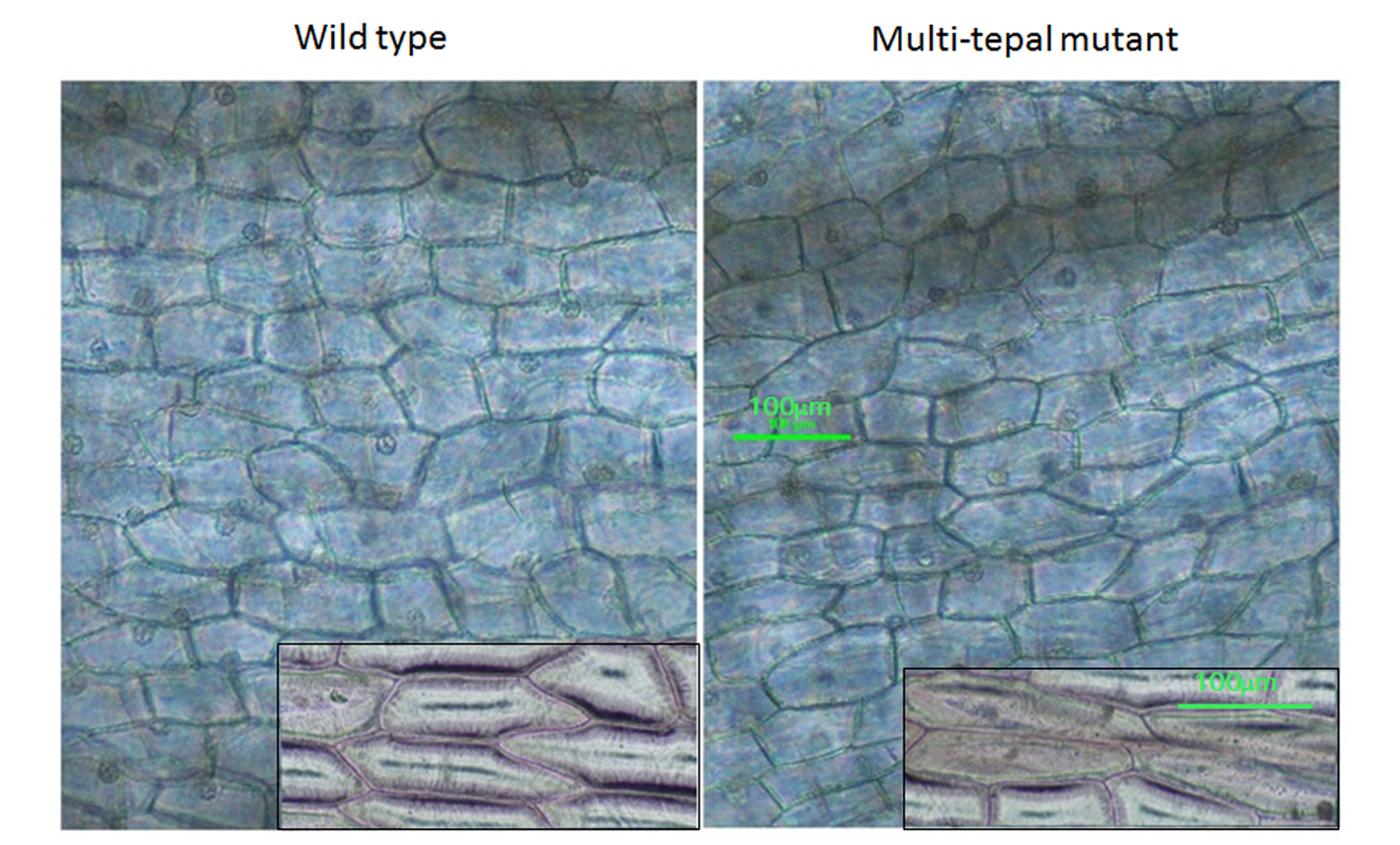

Supplement: Supplementary file 1 — Paradermal view of the epidermal cells of the petal both in the wild type and the mutant. (TIF 1951 kb) [file 12864_2017_3756_MOESM1_ESM.tif]

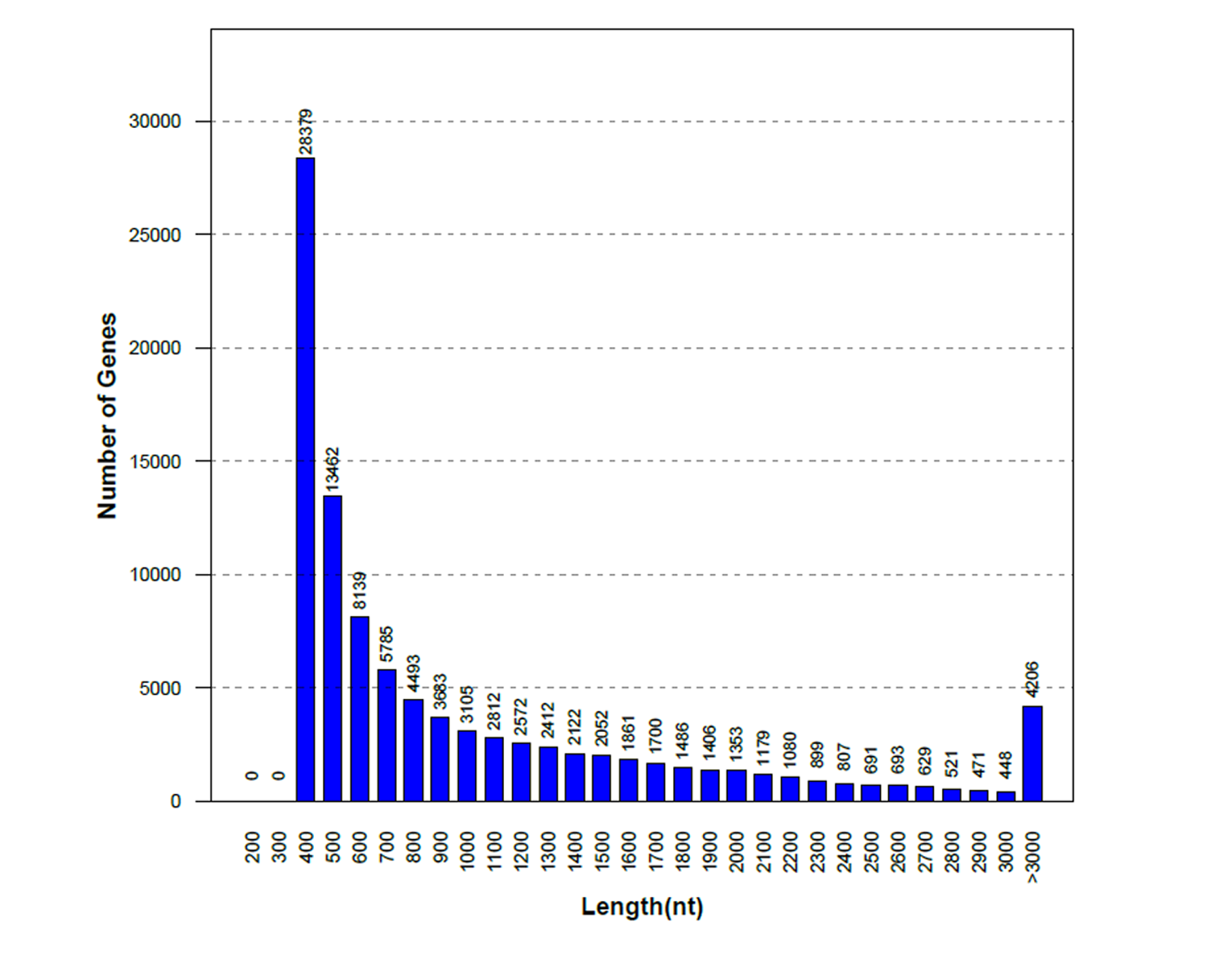

Supplement: Supplementary file 2 — The length distribution of assembled unigenes. The x-axis represents the sequence length in base pairs. The y-axis represents the unigenes number. (TIF 478 kb) [file 12864_2017_3756_MOESM2_ESM.tif]

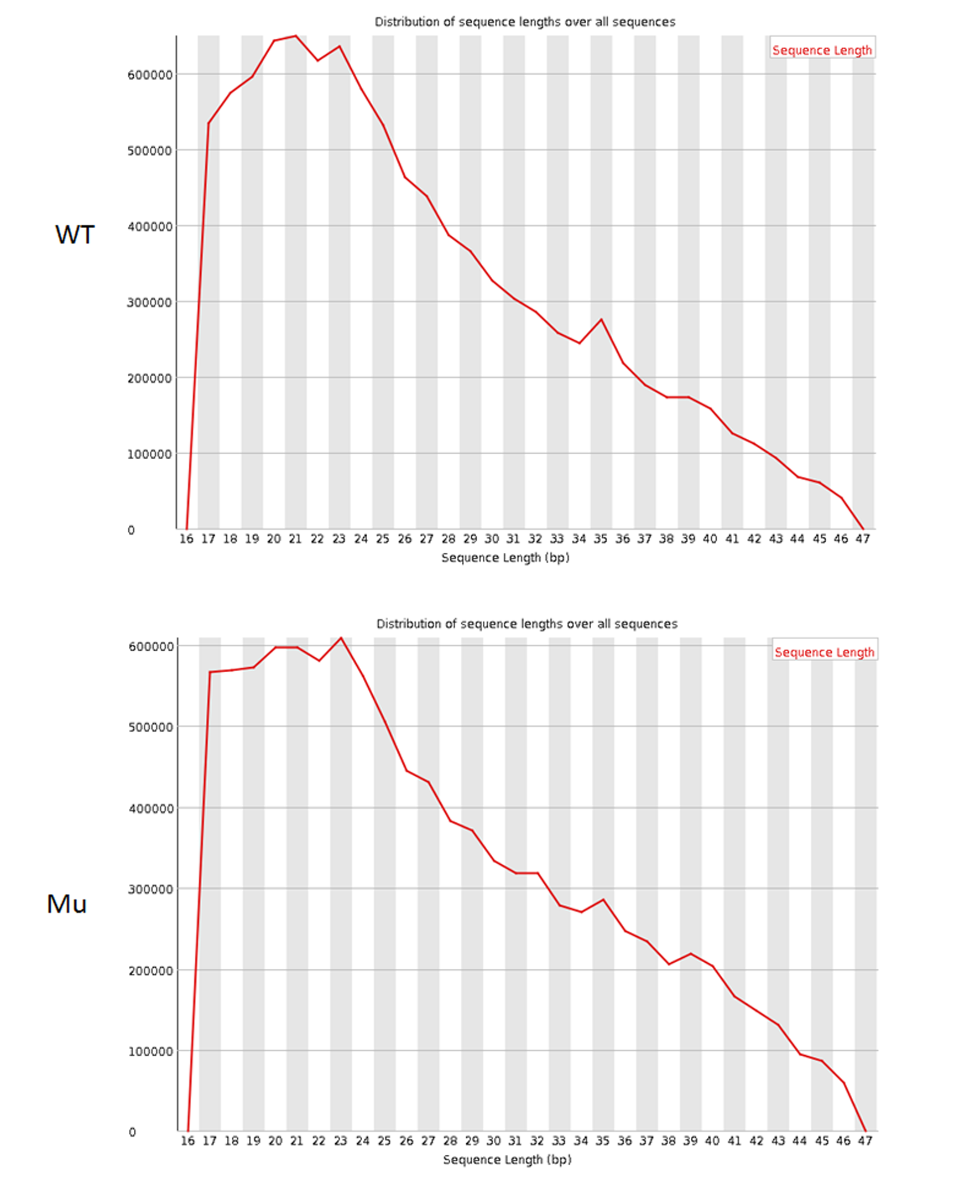

Supplement: Supplementary file 8 — Length distribution of small RNAs derived from Cymbidium goeringii ‘Songmei’ (WT) and the multi-tepal mutant ‘Yuhudie’ (Mu). (TIF 1182 kb) [file 12864_2017_3756_MOESM8_ESM.tif]

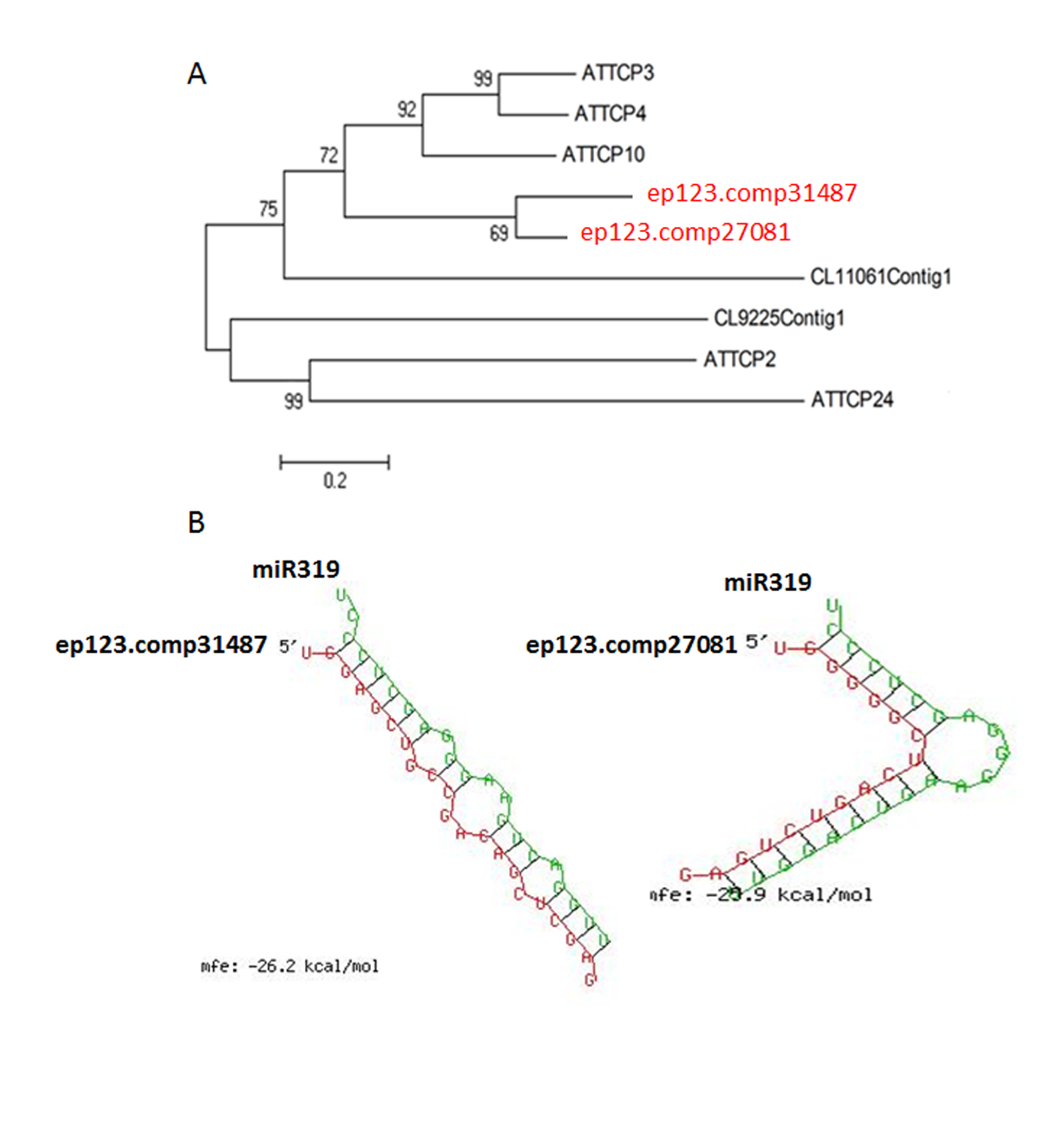

Supplement: Supplementary file 12 — Phylogenetic analysis of Cymbidium goeringii TCP–like genes with their homologues in Arabidopsis and the sequence complementary to miR396 using RNA hybrid software (http://bibiserv.techfak.uni-bielefeld.de/rnahybrid) (TIF 496 kb) [file 12864_2017_3756_MOESM12_ESM.tif]
