# Supplementary material for: Integrated mRNA and microRNA transcriptome variations in the multi-tepal mutant provide insights into the floral patterning of the orchid Cymbidium goeringii
Source: BMC Genomics. 2017 May 11;18:367. doi: 10.1186/s12864-017-3756-9 (PMC5426072; doi:10.1186/s12864-017-3756-9)
Supplement: Supplementary file 14 — The primers of stem-loop RT-PCR and qRT-PCR. (DOC 37 kb) [file 12864_2017_3756_MOESM14_ESM.doc]

Additional file 14. The primers of stem-loop RT-PCR and qRT-PCR

| **miRNA** | **Primers** |
| --- | --- |
| **miR396** | RT: GTCGTATCCAGTGCAGGGTCCGAGGTATTCGCACTGGATACGAAAGTTCAA  F: GTCAGCATTCCACAGCTTTC  R: TCGTATCCAGTGCAGGGTC |
| **miR319b** | RT: GTCGTATCCAGTGCAGGGTCCGAGGTATTCGCACTGGATACGAGGGAGCTC  F: GCATTGGATTGGACTGAAGG  R: TCGTATCCAGTGCAGGGTC |
| **SnRU6** | F: TCTAACAGTGTAGTTTGTCCCTTCG  R: TTGTGCGTGTCATCCTTGC |

| **miRNA** | **targets** | **Primers** |
| --- | --- | --- |
| miR319 | ep123.comp31487 | F:TCCTCGGTCGTATCGAAGGT  R:GTCGGACGTCGATGAGAAGG |
|  | ep123.comp27081 | F:AATTTTGGGTTGCGGTGTCG  R:TTGGGGTTTGGTCATCCATT |
| miR396 | ep456.comp59964 | F:GGCTTTCCCTATTCAAGCACAA  R:CCTCCACTTCTTGCCATCTGT |
|  | ep123.comp 17494 | F:CTGGGTTTTGGGCAGTTTCG  R:CCGGCTTCTACAGGCTTTCT |
|  | ep456.comp55669 | F:TATTCCAGTCCCTCCCGAGC  R:GTCAGGGTACGCGTCTTTTG |
|  | CL10167 | F:CACTAACACGACTGGGGTGG  R:TGATGCCGATTGTGAGGGAG |
